# Genome-scale identification and characterization of moonlighting proteins

**DOI:** 10.1186/s13062-014-0030-9

**Published:** 2014-12-11

**Authors:** Ishita Khan, Yuqian Chen, Tiange Dong, Xioawei Hong, Rikiya Takeuchi, Hirotada Mori, Daisuke Kihara

**Affiliations:** Department of Computer Science, Purdue University, 305 North University Street, West Lafayette, IN 47907 USA; Department of Biological Sciences, Purdue University, 240 Martin Jischke Drive, West Lafayette, IN 47907 USA; Graduate School of Biological Sciences, Nara Institute of Science and Technology, 8916-5, Takayama, Ikoma, Nara, 630-0192 Japan

**Keywords:** Moonlighting protein, Multitasking, Function annotation, Genome, Omics data

## Abstract

**Background:**

Moonlighting proteins perform two or more cellular functions, which are selected based on various contexts including the cell type they are expressed, their oligomerization status, and the binding of different ligands at different sites. To understand overall landscape of their functional diversity, it is important to establish methods that can identify moonlighting proteins in a systematic fashion. Here, we have developed a computational framework to find moonlighting proteins on a genome scale and identified multiple proteomic characteristics of these proteins.

**Results:**

First, we analyzed Gene Ontology (GO) annotations of known moonlighting proteins. We found that the GO annotations of moonlighting proteins can be clustered into multiple groups reflecting their diverse functions. Then, by considering the observed GO term separations, we identified 33 novel moonlighting proteins in *Escherichia coli* and confirmed them by literature review. Next, we analyzed moonlighting proteins in terms of protein-protein interaction, gene expression, phylogenetic profile, and genetic interaction networks. We found that moonlighting proteins physically interact with a higher number of distinct functional classes of proteins than non-moonlighting ones and also found that most of the physically interacting partners of moonlighting proteins share the latter’s primary functions. Interestingly, we also found that moonlighting proteins tend to interact with other moonlighting proteins. In terms of gene expression and phylogenetically related proteins, a weak trend was observed that moonlighting proteins interact with more functionally diverse proteins. Structural characteristics of moonlighting proteins, i.e. intrinsic disordered regions and ligand binding sites were also investigated.

**Conclusion:**

Additional functions of moonlighting proteins are difficult to identify by experiments and these proteins also pose a significant challenge for computational function annotation. Our method enables identification of novel moonlighting proteins from current functional annotations in public databases. Moreover, we showed that potential moonlighting proteins without sufficient functional annotations can be identified by analyzing available omics-scale data. Our findings open up new possibilities for investigating the multi-functional nature of proteins at the systems level and for exploring the complex functional interplay of proteins in a cell.

**Reviewers:**

This article was reviewed by Michael Galperin, Eugine Koonin, and Nick Grishin.

**Electronic supplementary material:**

The online version of this article (doi:10.1186/s13062-014-0030-9) contains supplementary material, which is available to authorized users.

## Background

With the overwhelming growth of genome sequence data produced by rapidly advancing sequencing technologies, the challenge of correctly determining functions of encoded proteins becomes ever more evident. As the number of functionally characterized proteins increases, it has been observed that there are proteins involved in more than one function [1-3]. These proteins were described as “moonlighting” proteins [1]. A moonlighting protein demonstrates multiple autonomous and usually unrelated functions. Diversity of dual functions of these proteins is in principle not a consequence of gene fusions, splice variants, multiple proteolytic fragments, homologous but non-identical proteins, or varying post-transcriptional modification.

The first and the most widely known example of moonlighting proteins was identified by Piatigorsky and Wistow [4] who showed that crystallins, structural proteins in the eye lens, also have enzymatic activity. Crystallins in several mammals, geckos, birds, and some other species are eye lens proteins that retain their metabolic functions, including lactate dehydrogenase, arginosuccinate lyase, and α-enolase [5-8]. Many known moonlighting proteins were originally recognized as enzymes, but there are also others that were known as receptors, channel proteins, chaperone proteins, ribosomal proteins, and scaffold proteins [1,9,10]. The secondary/moonlighting functions of these proteins include transcriptional regulation, receptor binding, apoptosis-related, and other regulatory functions. A variety of causes have been found for the moonlighting activities of these proteins [1], including locations inside and outside of cell (e.g. thymidine phosphorylase [11]), different locations within a cell (put A proline dehydrogenase [12]), ligand binding sites (*E. coli* aspartate receptor [13]), oligomerization states (glyceraldehyde-3-phosphate dehydrogenase [14]), differential expressions (neuropilin [15]), and ligand concentration (aconitase [16]).

As long as the additional functions do not interfere with the primary function, moonlighting functions can benefit a cell in several ways. Especially in prokaryotes, existence of multifunctional proteins aids in saving energy in cell growth and reproduction and makes their genomes more compact. Moonlighting proteins can also help in coordinating cellular activities in signalling pathways, transport, biosynthesis, and other functions [17]. It has been suggested that the presence of moonlighting proteins is under positive selection [1,10,18].

Recent papers [10,19] indicate that a number of moonlighting proteins in mammals play important roles in cellular activities and biochemical pathways that are involved in cancer and other diseases. Sriram et al. discussed how moonlighting functions may contribute to the complexity of metabolic disorders [20]. The positive selective pressure for developing moonlighting functions and the cell-level benefits given by moonlighting proteins suggest that the existence of moonlighting proteins in diverse genomes might be a common phenomenon.

Moonlighting proteins also pose a significant challenge to computational protein function annotation as current methods do not explicitly consider the possibility of dual functions for a protein. Conventional sequence-based functional annotation methods that are based on the concept of homology [21] or conserved motifs/domains [22-24] will have problems for identifying secondary functions because there are cases that a homolog of a moonlighting protein does not possess the secondary function [25] or has a different secondary function [16,26,27]. There are two studies that have investigated whether existing sequence-based function prediction methods can identify distinct dual functions of moonlighting proteins [28,29]. Gomez et al. compared eleven methods and reported that PSI-BLAST [21] performed relatively well in identifying moonlighting functions [28]. We have compared our function prediction tools, PFP [30,31] and ESG [32], with PSI-BLAST and showed that PFP, which mines function information from weakly similar sequences, had the best performance in predicting two distinct functions of moonlighting proteins [29]. These two studies suggest that secondary functions may be found in distantly related sequences if not in close homologs; however, further investigation is needed because the studies are based on a limited dataset. Gomez et al. have also analyzed protein-protein interactions (PPIs) of moonlighting proteins and showed that GO terms of secondary function are enriched in interacting proteins, although they concluded that predicting correct secondary function from a PPI network is not an easy task [33]. Computational works on moonlighting proteins were recently summarized in a review article [34].

Despite the potential abundance of moonlighting proteins in various genomes and their important roles in pathways and disease development, systematic studies of moonlighting proteins are still in their early stage for obtaining a comprehensive picture of proteins’ moonlighting functions and also for developing computational methods for predicting moonlighting proteins. The limited number of known moonlighting proteins is mainly because secondary functions of proteins are usually found unexpectedly by experiments. To lay the foundation for studying moonlighting proteins, the current work is aimed at establishing a framework for systematically identifying moonlighting proteins in an organism using currently available function annotations and omics-scale data. This work consists of two logical parts. First, we examined Gene Ontology (GO) annotations [35,36] of known moonlighting proteins in the UniProt protein sequence database [37] to see if functional diversity of moonlighting proteins is reflected in current GO annotations. Since the systematic study of moonlighting proteins is still in an early stage, most of the cases they are not explicitly labelled in the database as “moonlighting”, “dual function”, “multitasking”, or related words, which makes it difficult to collect and reuse existing knowledge of moonlighting proteins. We analyzed the GO terms assigned to each known moonlighting protein and found that the GO term semantic similarity score can clearly separate the GO terms of the diverse functions of these proteins. Encouraged by this result, we further analyzed the GO term annotations of protein genes in the *Escherichia coli* K-12 genome and found 33 novel moonlighting proteins by identifying genes with clear GO term separations. We confirmed in literature that the dual functions of the identified proteins had experimental evidence. Among our computationally identified moonlighting proteins, we later found that DegP was experimentally identified as a moonlighting protein with both protease and chaperone activity [38-40], which confirmed that our procedure was valid.

In the second part of this work, we investigated characteristics of moonlighting proteins in omics-scale data, namely, protein-protein interaction, gene expression, phylogenetic profile [41], and genetic interactions [42]. We decided to analyze these omics-scale data because moonlighting proteins’ distinct functions may display characteristic features in association patterns with other proteins. In analyzing protein-protein interactions, we found that moonlighting proteins interact with a higher number of distinct functional classes of proteins than non-moonlighting ones, which intuitively stems from the functional diversity of these proteins. We found a substantial number of moonlighting proteins in the PPI network of moonlighting proteins, suggesting moonlighting proteins tend to interact with other moonlighting proteins. It is also notable that moonlighting proteins share their primary functions with the majority of interacting proteins. Similarly, a weak tendency was found that moonlighting proteins interact with proteins from more diverse functional classes in gene expression and phylogenetic profile networks. We have further examined structural features of proteins, i.e. ligand binding sites and disordered regions. We analysed disordered regions and found that a larger fraction of moonlighting proteins have intrinsically disordered regions than non-moonlighting proteins. Finally, although there are only a few moonlighting proteins whose tertiary structures were available, we found cases where the binding sites that correspond to distinct functions are located in separate regions of the proteins’ tertiary structures.

## Results

### Pairwise GO semantic similarity analysis

We investigated whether the distinct dual functions of moonlighting proteins were reflected in their GO term annotations. We used 58 experimentally confirmed moonlighting proteins in three datasets (see Methods). We classified the GO terms of these proteins into four classes: GO terms that belong to the “primary” function of the protein (Function 1, F1), terms that belong to the “secondary” function (Function 2, F2), terms that belong to both functions (F3), and terms that do not belong to either of the functions. For each moonlighting protein, we computed the relevance semantic similarity score (*SS*^*Rel*^, Eqn. 1) for three types of GO term pairs: pairs where both terms belong to either F1 or F2 and pairs that consist of one GO term from F1 and the other from F2. *SS*^*Rel*^ ranges from 0.0 to 1.0 with 0.0 for the least similarity and 1.0 for the highest similarity.

**Figure 1:**
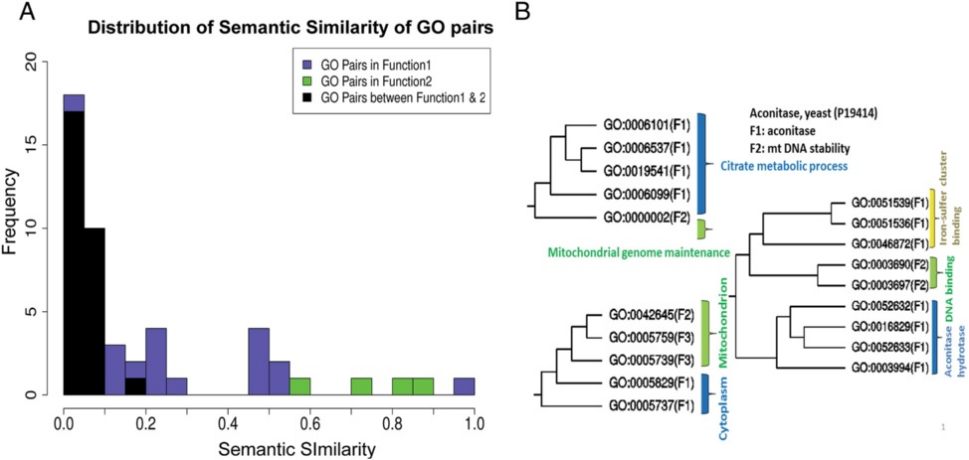
**Semantic similarity distribution.** The distribution of the relevance semantic similarity *SS* ^*Rel*^ score of GO term pairs, aconitase, yeast (Uniprot ID : P19414). **(A)** *SS* ^*Rel*^ distribution of GO pairs within the primary function (function 1), the secondary function (function 2), and pairs from function 1 and 2. **(B)** Hierarchical clustering of GO terms in the three GO categories using pairwise *SS* ^*Rel*^ scores.

Figure 1 shows an example of the semantic similarity of GO pairs for aconitase in yeast (UniProt ID: P19414). This protein was initially identified as an enzyme in the tri-carboxylic acid (TCA) cycle, which catalyzes the isomerization of citrate to iso-citrate via cis-aconitate. The GO terms for F1 include TCA cycle (GO:0006099), propionate metabolic process (GO:0019541), glutamate biosynthetic process (GO:0006537), citrate metabolic process (GO:0006101), cytosol (GO:0005829), cytoplasm (GO:0005737), citrate hydro-lyase (GO:0052632), lyase activity (GO:0016829), iso-citrate hydro-lyase (GO:0052633) and aconitate hydratase activity (GO:0003994). The enzyme’s secondary function (F2) was later found as a “role in mitochondrial DNA maintenance” [26], which is annotated with GO terms including mitochondrial genome maintenance (GO:0000002), mitochondrial nucleoid (GO:0042645), single-stranded-DNA binding (GO:0003697), and double-stranded-DNA binding (GO:0003690). The GO terms that belong to both the primary and secondary functions (F3) are “mitochondrion” and “mitochondrial matrix” (GO:0005759). Figure 1A shows the *SS*^*Rel*^ score distribution of GO term pairs, those within F1 or F2 and pairs across F1 and F2 (F1F2 pairs). It is apparent that the *SS*^*Rel*^ scores for all the F1F2 pairs are very small, below 0.2. All four F2 pairs have large scores over 0.4. As for F1 pairs, 8 out of 27 have large scores over 0.4. We must note that 12 F1 pairs have a score of 0, which occurs when the lowest common ancestor for a GO term pair is at the root of the GO hierarchy. In the case of aconitase, the majority of the 0 scores for F1 pairs occurred between terms related to ion-sulfur cluster binding and aconitase hydrolase (Figure 1B).

Figure 1B shows a hierarchical clustering of GO terms of aconitase based on *SS*^*Rel*^. In all three GO categories, terms in F1 and F2 were clearly separated. In the Biological Process (BP) ontology, the only GO term in F2 is “mitochondrial genome maintenance” (GO:0000002), which is separated from the other F1 GO terms. In the Molecular Function (MF) ontology, the GO terms with F2 labels (ssDNA and dsDNA binding, GO:0003697 and GO:0003690, respectively) form a cluster that is separate from the F1 GO terms. Two separate clusters were formed for F1 terms in MF, “Iron-Sulfer cluster binding” GO terms (highlighted in yellow) and terms related to aconitase enzymatic activity. The former F1 cluster lies closer to the F2 cluster due to a common ancestral term “binding”. In the Cellular Component (CC) ontology, the F2 term “mitochondrial nucleoid” (GO:0042645) is separate from F1 GO terms (related to cytoplasm) but clustered with two F3 terms.

**Figure 2:**
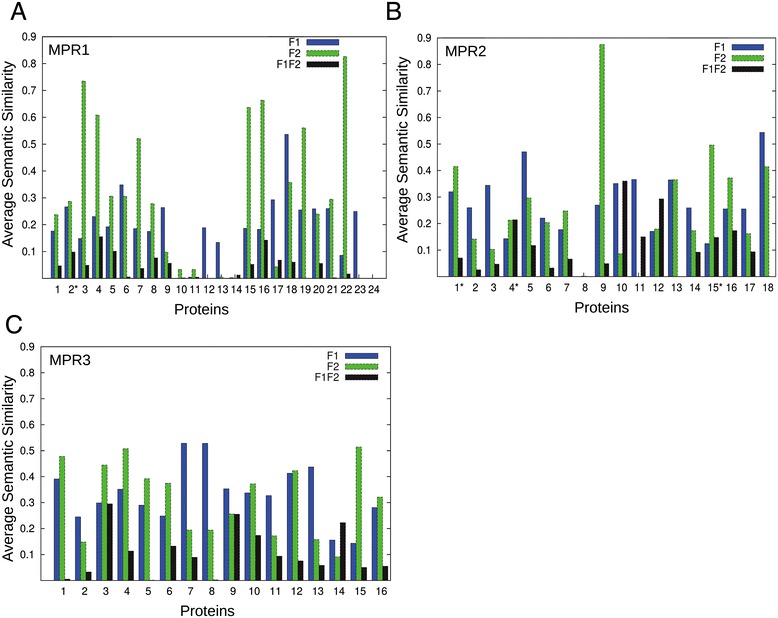
**Average** ***SS*** ^***Rel***^ **of GO term pairs for moonlighting proteins.** Average *SS* ^*Rel*^ of GO pairs within function 1, function 2, and pairs from function 1 and 2 were computed separately. **(A)** Moonlighting proteins in the MPR1 set. Protein 24 is presenilin in *Physcomitrella patens* (Uniprot ID: A9S846). This protein have one GO term each in F1 and F2 (F1 term GO:0004190, “aspartic type endopeptidase activity” and F2 term GO:0016021, “intergral to membrane”). The two GO terms are in different ontologies, MF and CC respectively, and thus the scores are zero for F1 and F2 (because there is only one term) as well as F1-F2 (because similarity of GO terms in different categories cannot be considered). **(B)** the MPR2 set; and **(C)** the MPR3 set.

Next, we show the mean *SS*^*Rel*^ score for GO pairs within F1 or F2 and across F1 and F2 for all moonlighting proteins in the three datasets (Figure 2). The mean *SS*^*Rel*^ scores for F1 pairs and F2 pairs are higher than those for across F1F2 pairs in 51 (87.9%) moonlighting proteins (MPR1-3 datasets). One exception of this trend is Protein 17 in MPR1 (Figure 2A). This protein is aconitase of *Mycobacterium tuberculosis* (UniProt ID: O53166), which has “TCA cycle enzyme” as F1 and “iron-responsive protein” as F2. This protein switches between the two functions depending on the cellular iron levels, namely, binding of a 4Fe-4S cluster occurs as a part of the aconitase function whereas binding of a 3Fe-4S cluster triggers the secondary function [16]. Thus, the GO term for “4 iron, 4 sulfur cluster binding” (GO:0051539) was classified for F1 and “3 iron, 4 sulfur cluster binding” (GO:0051538) for F2, which resulted in a relatively high *SS*^*Rel*^ score of 0.698 for this F1F2 pair.

**Figure 3:**
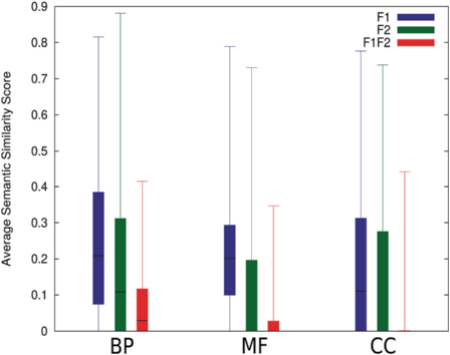
**Average** ***SS*** ^***Rel***^ **distribution.** Box-and-whisker plots for average *SS* ^*Rel*^ distribution of BP, MF, and CC GO pairs for the moonlighting proteins in the MPR1-3 sets excluding proteins with * in Figure 2. The top and the bottom of a box show the first and third quartiles and the line in the middle of a box is the median. The two ends of whisker show the minimum and the maximum values.

Figure 3 summarizes the distribution of the average *SS*^*Rel*^ score for F1, F2, and F1F2 GO pairs in the BP, MF, and CC ontologies for the proteins in MPR1-3. The Friedman test was performed to evaluate statistical significance of score difference between F1, F2, and F1F2 GO term pairs. It was shown that the F1F2 pairs have significantly smaller scores than F1 and F2 pairs in BP and CC (p-value < 0.05). As for MF, the score difference of F1F2 pairs from F1 pairs had a p-value below 0.05 but the p-value versus F2 pairs was a slightly larger value of 0.097.

### Moonlighting and non-moonlighting proteins in *E. coli*

The previous section showed that GO terms of moonlighting proteins can be clustered into distinct functions using the *SS*^*Rel*^ score. In this section we identified potential moonlighting proteins in the *Escherichia coli* K-12 genome by examining clusters of GO term annotations taken from UniProt. We used GO terms of the BP ontology because BP GO terms showed a clearer separation between F1 and F2 functions (Figure 3).

**Figure 4:**
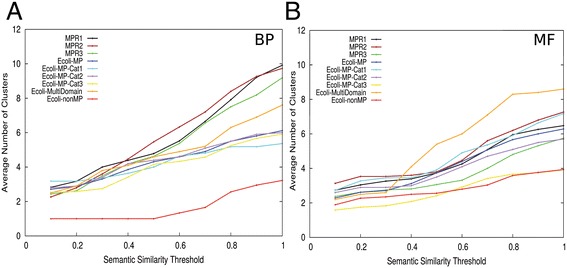
**Clustering profiles of sets of moonlighting and non-moonlighting proteins.** For each protein in a dataset, GO terms were clustered using various threshold values of *SS* ^*Rel*^ and average number of GO term clusters were plotted. The datasets plotted were experimentally known moonlighting proteins (MPR1, 2, and 3) and identified moonlighting and non-moonlighting proteins in *E. coli* (Ecoli-MP and Ecoli-nonMP). *E. coli* moonlighting proteins were also plotted separately for each evidence category, 1 to 3 (Ecoli-PosMP-Cat1-3; see Methods) as well as multi-domain multi-function proteins. **(A)** BP GO terms were considered. **(B)** MF GO terms were considered.

Figure 4 shows clustering profiles of moonlighting proteins, where GO terms in BP and MF (Figure 4A and B) were clustered using single linkage clustering at different *SS*^*Rel*^ cutoff values. A clustering profile provides a more thorough picture of GO term similarities than clustering using a single cutoff value. It can show how the number of clusters grows at different cutoff values. Using the profiles for moonlighting proteins in MPR1 (black), MPR2 (red), and MPR3 (green) as a reference, the following three criteria were used to identify potential moonlighting proteins in *E. coli*: 1) proteins that have at least eight GO terms in the UniProt annotation; 2) proteins that have at least two clusters in the clustering profile at a *SS*^*Rel*^ cutoff of 0.1; 3) proteins that have at least four clusters in the clustering profile at a 0.5 *SS*^*Rel*^. 140 proteins were found to satisfy all of these three criteria. We have also identified potential non-moonlighting proteins by applying essentially the opposite criteria to above: 1) proteins that have at least eight GO terms in the UniProt annotation; 2) proteins that have at most one cluster at a *SS*^*Rel*^ of 0.1; 3) proteins that have at most one cluster at 0.5 *SS*^*Rel*^. There were 150 proteins that satisfied these criteria for non-moonlighting proteins.

For the 140 identified potential moonlighting proteins, we manually consulted original literature to determine the level of experimental support for annotated functions and whether diverse functions are directly related to each other. This literature check step has selected 43 proteins that have distinct dual functions. Subsequently, we used the Pfam database [22] to find domains in the 43 proteins in order to distinguish proteins whose multi-functionality originates from different domains. GO terms associated with each Pfam domain in a protein were compared with the primary and secondary functions of the protein. Finally, 33 proteins were selected as moonlighting proteins through this post-processing (Table 1). The selected moonlighting proteins were further classified into three categories. The first category is for moonlighting proteins that have clear experimental evidence for two independent functions. The second category is proteins for which we found literature evidence of two diverse functions, but no evidence was found as to whether those two functions are independent or related. The third category is for “weak” moonlighting proteins for which the evidence for the secondary function was found from a large scale assay or a phenotypic experiment of mutants and the relationship between the primary and the newly found secondary function is not known. We would like to note that some of the moonlighting proteins classified into the second or the third category are so-called neomorphic moonlighting proteins [19], which exhibit the secondary function due to a mutation or conformational change.

**Table 1:** **Moonlighting proteins identified in** ***E. coli***

**Proteinname/uniprot ID/gene ID**	**First function**	**Additional functions**	**Category** ^**a)**^	**Ref.**
b0118/P36683/AcnB	Aconitate hydratase	Post-transcriptional regulation; mRNA binding	I	[27]
b1019/P31545/EfeB	Peroxidase on guaiacol	Iron assimilation from heme; response to DNA damage stimulas	I	[43]
b1276/P25516/AcnA	Aconitate hydratase	Post-transcriptional regulation; mRNA binding	I	[27]
b1967/P31658/HchA	Molecular chaperone	Glyoxalase activity	I	[44]
b3183/P42641/ObgE	GTPase	Role in ribosome biogenesis	I	[45,46]
b4151/P0A8Q3/FrdD	Membrane bound respiratory protein (anaerobic condition)	Role in bacterial flagellar switch (aerobic conditions)	I	[47]
b4152/P0A8Q0/FrdC	Membrane bound respiratory protein (anaerobic condition)	Role in bacterial flagellar switch (aerobic conditions)	I	[47]
b4153/P0AC47/FrdB	Membrane bound respiratory protein (anaerobic condition)	Role in bacterial flagellar switch (aerobic conditions)	I	[47]
b4154/P00363/FrdA	Membrane bound respiratory protein (anaerobic condition)	Role in bacterial flagellar switch (aerobic conditions)	I	[47]
b4179/P21499/Rnr	Helicase	RNase	I	[48]
b4260/P68767/PepA^†b)^	Plasmid recombination	Peptide catabolic process; DNA binding/transcriptional control	I	[49]
b0161/P0C0V0/DegP^†^	Chaperone	Proteolysis	II	[50]
b0509/P77161/GlxR	Glyoxylate metabolism	Allantoin assimilation; DNA damage response	II	[51,52]
b0957/P0A910/OmpA	Transport	1. Viral entry 2.DNA damage response	II	[51,53]
b1317/P77366/YcjU	Carbohydrate metabolism	1. Cell-to-cell plasmid transfer 2. Reduce the lethal effects of stress	II	[54,55]
b1710/P06610/BtuE	Glutathione peroxidase	Non-essential role in vitamin-B12 transport	II	[56,57]
b2415/P0AA04/PtsH	Phosphocarrier protein essential in sugar transport	Positive regulation of glycogen catabolism	II	[58]
b2552/P24232/Hmp	(aerobic condition) Nitric oxide dioxygenase (NOD)	(anaerobic condition) Amplifier of superoxide stress, NO and FAD reductase	II	[59,60]
b2949/P0A8I1/YqgF	Putative Holliday junction resolvase	Transcription anti-termination	II	[61,62]
b3414/P63020/NfuA	Fe-S biogenesis	Necessary for the use of extracellular DNA as the sole source of carbon and energy	II	[63]
b3463/P0A9R7/FtsE	Cell division	Salt transport by ABC-Transporter	II	[64]
b3706/P25522/MnmE	tRNA modification	Regulating glutamate-dependent acid resistance	II	[65]
b0135/P31058/YadC	Cell adhesion	Reduce lethal effects of stress	III	[55]
b0284/P77489/YagR	Putative xanthine dehydrogenase	DNA damage response	III	[51]
b0543/P23895/EmrE	Multidrug transporter	DNA damage response	III	[51]
b1018/P0AB24/EfeO	Involved in Iron uptake	Response to lethal antimicrobial and environmental stress	III	[55]
b2037/P37746/RfbX	Putative O-antigen transporter	DNA damage response	III	[51]
b2147/P25889/PreA	Pyrimidine base degradation	Required for swarming motility	III	[66]
b2290/P0A959/AlaA	Involved in biosynthesis of alanine	Response to lethal antimicrobial and environmental stress	III	[55]
b3191/P64602/MlaB	Phospholipid ABC transporter	Response to lethal antimicrobial and environmental stress	III	[55]
b3233/P0A9Q9/Asd	Aspartate-semialdehyde dehydrogenase	DNA damage response	III	[51]
b4177/P0A7D4/PurA	Adenylosuccinate synthetase	DNA damage response	III	[51]
b4383/P0A6K6/DeoB	Phosphopentomutase	DNA damage response	III	[51]

^a)^Moonlighting proteins are classified into four categories: I, both primary and the secondary functions have clear experimental evidences that they are independent; II, both primary and the secondary functions have experimental evidences but it is not clear if the functions are independent; III, “weak” moonlighting proteins, evidences for the secondary function is from a large scale assay or a phenotypic experiment of mutants and the relationship between the primary and the secondary function is not known. Proteins are sorted by the b number within each category.

^b)^Proteins included in either MoonProt or MultiTaskDB are indicated with^†^. PepA is included in the MoonProt database. DegP is included in both MoonProt and MultiTaskDB.

Table 2 lists ten multi-functional and multi-domain proteins that were excluded by the Pfam domain search from the final list of moonlighting proteins. These proteins happen to include five multi-reaction enzymes, which are enzymes that are generally listed as bi-functional or multi-functional proteins in UniProt and in literature. They perform multiple reactions with similar substrates in the same or different pathways. A multi-reaction enzyme is not included as a moonlighting protein in the original definition [18]. However, they are kept here along with the five other multi-domain proteins in Table 2 because they were detected by the GO clustering criteria.

**Table 2:** **Multi-domain proteins with multiple functions identified in** ***E. coli***

**Gene ID /Protein name/uniprot ID**	**First function**	**Additional functions**	**Ref.**
b0002/P00561/ThrA	Aspartokinase	Homoserine dehydrogenase	[67]
b0529/P24186/FolD	Oxidation of methylenetetrahydrofolate	Hydrolysis of methenyltetrahydrofolate	[68]
b1241/P0A9Q7/AdhE	Alcohol dehydrogenase	Acetaldehyde dehydrogenase; Pyruvate-formate-lyase deactivase	[69,70]
b1888/P07363/CheA	Chematoxis sensor kinase	Regulation of protein; dephosphorylation	[27,71,72]
b2255/P77398/ArnA	Oxidative decarboxylation of UDP-glucuronic acid	Formyltransferase	[73]
b3052/P76658/HldE	D-beta-D-heptose 7-phosphate kinase	D-beta-D-heptose 1-phosphate adenosyltransferase	[74]
b3368/P0AEA8/CysG	SAM-dependent methylation	NAD-dependent ring dehydrogenation; Ferrorochelation	[75]
b3650/P0AG24/SpoT	ppGpp synthase	ppGpp hydrolase	[76,77]
b3940/P00562/MetL	Aspartokinase	Homoserine dehydrogenase	[67]
b4390/P27278/NadR^†^	Transcriptional regulator	Nicotinamide mononucleotide adenylyltransferase; Ribosylnicotinamide kinase	[78]

^†^This protein is included in MoonProt.

The identified 33 moonlighting proteins (Table 1) and 10 multi-domain multi-function proteins (Table 2) do not have many overlap with the MoonProt database [79] and MultitaskProtDB [80]. Only two (PepA and DegP) in Table 1 and one (NadR) in Table 2 were found in the two databases.

Among the 140 proteins that were identified by the GO clustering criteria, 97 (69.3%) of them were discarded later by the literature survey. The discarded proteins satisfied the three GO term clustering criteria but either a) the sufficient number of GO term clusters was due to a non-descriptive GO term at a high (general) level of the GO hierarchy such as “transport” or “biosynthesis”, which resulted in a small similarity scores with the other GO terms; or b) experimental evidence of GO terms were found in literature only for one of its functions but not the other. Proteins discarded by the latter reason may be confirmed as moonlighting proteins in the future when experimental evidence is made available.

Clustering profiles of the identified moonlighting and non-moonlighting proteins in *E. coli* are shown in Figure 4 in comparison with the MPR1-3 datasets. Three categories of moonlighting proteins as well as multi-domain multi-functional proteins were also separately plotted. Clearly, the number of GO term clusters for moonlighting proteins is higher than non-moonlighting proteins for both BP and MF. In the MF ontology, the multi-domain multi-functional proteins have a larger number of clusters than the rest for high cutoff values of over 0.4. The two-sample Kolmogorov-Smirnov (KS) test showed that the *E. coli* moonlighting proteins (Ecoli-PosMP in Figure 4) and the MPR1-3 sets have significantly larger numbers of clusters than the *E. coli* non-moonlighting proteins (Ecoli-NegMP) at the three semantic similarity thresholds, 0.1, 0.5, and 1.0 for the BP ontology (Figure 4A) (p-values < 0.05). As for the MF ontology, *E. coli* moonlighting proteins have significantly larger number of clusters than the *E. coli* non-moonlighting proteins at threshold 1.0, using a p-value cutoff of 0.05. The full results of the KS tests are provided in (Additional file 1: Table S1).

It was noticed that known moonlighting proteins in the MPR1-3 sets have more GO annotations than the *E. coli* moonlighting proteins, which is a part of the reason why the MPR1-3 sets have more GO clusters (Figure 4). The average number of BP GO annotations of the *E. coli* moonlighting proteins was 5.76 while the MPR1-3 proteins had 9.65 terms. The clustering profile analysis can identify new moonlighting proteins from their existing GO annotations in UniProt. However, a limitation is that candidate proteins need to be well annotated with a sufficient number of GO terms. Indeed only 29.1% of *E. coli* proteins have eight or more GO terms and were subject to this analysis. In the subsequent sections, we will explore different ways to identify potential moonlighting proteins that do not require GO annotations.

### Protein-protein interaction network

From this section, we examine characteristic features of moonlighting proteins in large-scale omics data. We begin with the protein-protein interaction (PPI) network. Interacting proteins tend to share common function and thus a PPI network can be used as a valuable source for predicting protein function [81]. It was also shown that PPI networks are helpful in detecting additional novel function of well-known proteins [82]. We obtained physically interacting proteins from the STRING database [83].

**Figure 5:**
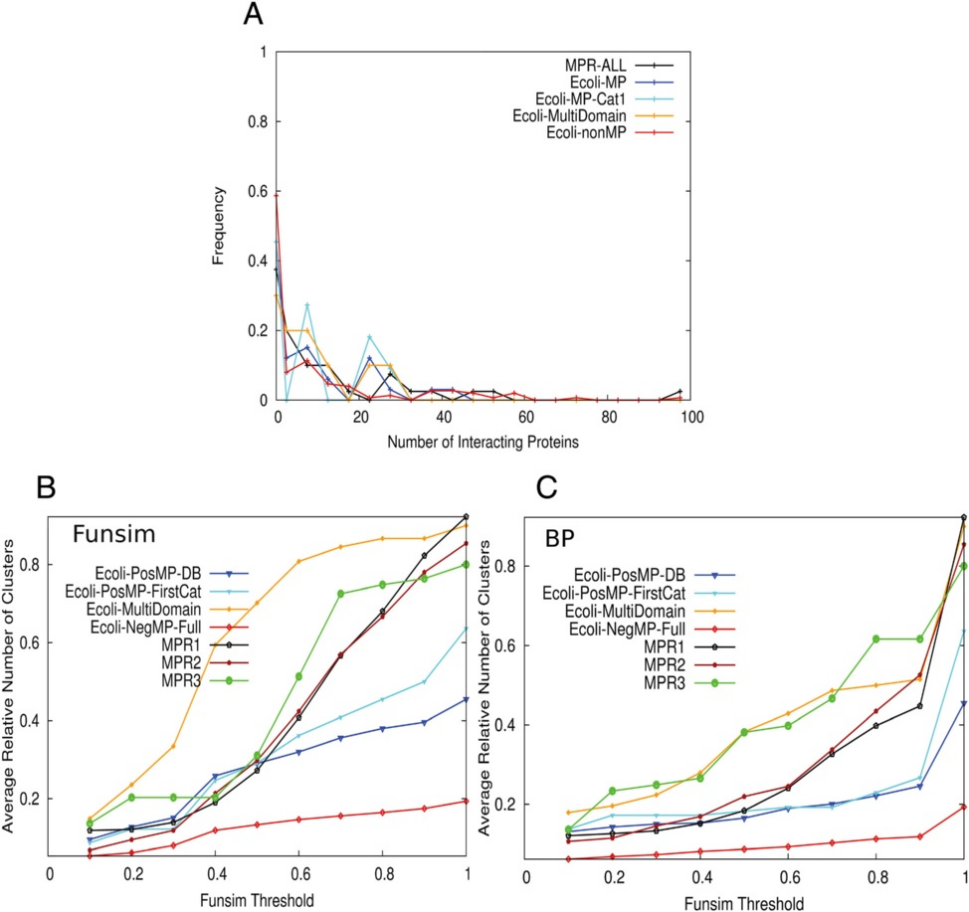
**Interacting proteins of moonlighting and non-moonlighting proteins.** Physically interacting proteins were obtained from the STRING database. **(A)** Histogram of the number of interacting proteins. Five datasets are shown: known moonlighting proteins in the MPR1-3 sets (MPR-ALL), the identified moonlighting proteins in *E. coli* (Ecoli-MP), moonlighting proteins detected in *E. coli* that have clear experimental evidences for the dual functions and classified into the category 1 (Ecoli-MP-Cat1), *E. coli* proteins whose multi-functionality originates from different domains (Ecoli-MultiDomain) and non-moonlighting proteins in *E. coli*. Values on the y-axis are the fraction of the proteins among the entire proteins in each dataset. The bin size used was five. **(B)**, average number of clusters of interacting proteins clustered using the funsim score (Eqn. 4). Seven datasets are plotted: MPR1, MPR2, MPR3, Ecoli-MP, Ecoli-MP-Cat1, Ecoli-MultiDomain, and Ecoli-nonMP. **(C)** Clustering was performed using the funsim score of BP terms only (Eqn. 3).

First, we examined the number of interacting proteins of moonlighting and non-moonlighting proteins (Figure 5A). In addition to the *E. coli* moonlighting and non-moonlighting proteins, histograms for the MPR1-3 sets are shown for comparison. Among the *E. coli* MP set, 11 proteins in the first category (those that have clear experimental evidence of their dual functions) were also separately plotted to verify that the observed trend for the entire *E. coli* MP set was consistent with its most reliable subset. Overall MP and nonMP have similar distributions with the largest peak at 0–5 interacting proteins. A small peak at 20–25 interacting proteins was observed for *E. coli* MP. This peak consists of two proteins, pepA (P68767) and frdB (P0AC47).

Next, we checked the functional divergence of interacting proteins. Using the same datasets as Figure 5A, interacting proteins for each moonlighting or non-moonlighting proteins in the datasets are clustered based on their functional similarity using the funsim score (Eqn. 4). In Figure 5B, the average numbers of clusters per interacting protein at different threshold values are plotted. The funsim score of all three GO categories was used for Figure 5B while the funsim score with only BP (BP-funsim score) was used for Figure 5C. In the two clustering profiles (Figures 5B & 5C) the non-MP set has consistently lower number of clusters as compared to moonlighting proteins. *E. coli* MPs and non-MPs show a clear contrast in the number of clusters with the former having over twice as many clusters as the latter. Consistent results were obtained when interacting proteins were selected from the STRING database using a score that combines different types of evidence including physical interactions, comparative genomics approaches, and gene expression (data not shown). A pairwise two-sample KS divergence test showed that the average number of clusters of the *E. coli* MP and nonMP sets is significantly different at the funsim-BP threshold values of 0.2, 0.6, and 0.8 and funsim threshold values 0.6 and 1.0 (Additional file 1: Table S1). To conclude, the results show that moonlighting proteins interact with proteins with more diverse functions than non-moonlighting ones.

### Do interacting proteins share moonlighting functions?

**Figure 6:**
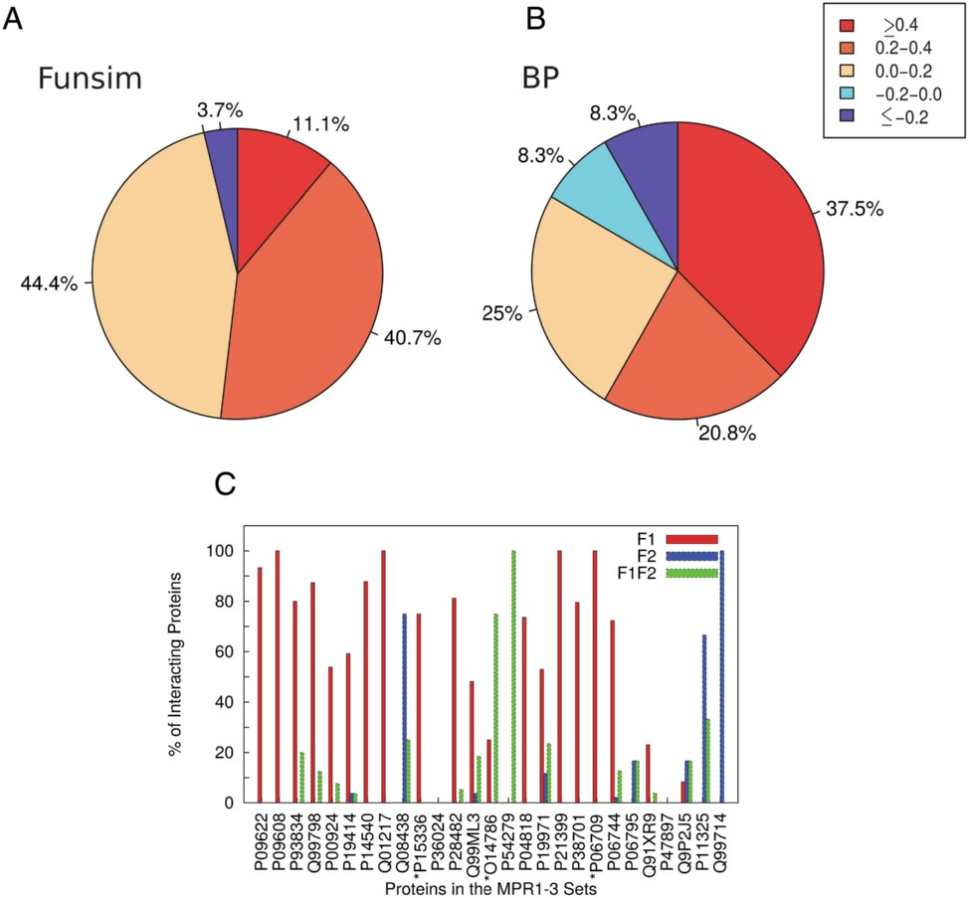
**Function similarity analysis.** Functional similarity between interacting proteins and the primary and secondary functions of moonlighting proteins. 27 moonlighting proteins in the MPR1-3 sets that have physically interacting proteins in STRING database and their 575 interacting proteins were analyzed. **(A)** The functional similarity score is computed between GO terms of the primary (F1) or the secondary (F2) functions of a moonlighting protein against the entire GO terms of its interacting protein and the score difference was computed. Interacting proteins were classified by the range of funsim score difference between F1 and F2 GO terms from their interacting moonlighting proteins. **(B)** The same type of chart as panel A, using the BP-funsim score. **(C)** For each moonlighting protein, percentages (%) of interacting proteins sharing F1, F2, or both functions of moonlighting proteins are shown. The BP-funsim score was used to determine if proteins share functional similarity. If an interacting protein has a BP-funsim score to both F1 and F2 GO terms of the moonlighting protein, it is classified as both. An interacting protein is considered to share F1, F2, or both functions if the BP-funsim score is larger than the mean *SS* ^*Rel*^ score of BP GO pairs of F1 or F2 in the moonlighting protein. In the case that a moonlighting protein has 0 *SS* ^*Rel*^ score, the cutoff was set to 0.4 for an interacting protein to be considered to share F1, F2, or both functions. P47897 does not have any interacting proteins with F1 or F2 function. Its only interacting protein, RSBN1, has a BP-funsim score of 0 with F1 and F2 functions of P47897. P36024 also does not have any interacting proteins sharing F1 or F2 function. Out of its four interacting proteins, YKL088W has the highest funsim-BP score with F1/F2 GO terms of P36024 (score 0.25), which is below the funsim-BP F1/F2 cutoff for P36024 (cutoff 0.4 for both F1 and F2).

We also investigated the extent to which the primary and secondary functions of a moonlighting protein are shared by its interacting proteins. For this analysis, we used 27 moonlighting proteins in the MPR1-3 sets that have interacting proteins because GO terms for their primary and secondary functions were manually classified. For each moonlighting protein in MPR1-3, we computed the functional similarity of its primary function (F1) and its secondary function (F2) separately against GO term annotation of its interacting proteins. Functional similarity was quantified by the funsim score (Figure 6A) and the BP-funsim score (Figure 6B). To determine if an interacting protein was biased to either the F1 or F2 function, the score difference between F1 and F2 was computed.

It is evident that the F1 function is dominant for the majority of the interacting proteins. When the funsim score was considered (Figure 6A), 96.3% of the interacting proteins had functions closer to the F1 rather than the F2 function. The dominance of F1-oriented functions in interacting proteins is consistent in Figure 6B, where the BP-funsim score was considered.

Figure 6C provides results for individual moonlighting proteins. For a moonlighting protein, GO terms of its F1 and F2 functions were compared separately to the entire GO annotation of each interacting protein. If GO terms of an interacting protein have a BP-funsim score that is larger than the mean *SS*^*Rel*^ scores of BP terms in F1 or F2 of the moonlighting protein, the interacting protein was considered to share common F1 or F2 function, respectively, with the moonlighting protein. In the case that a moonlighting protein has very diverse F1 or F2 GO terms in itself with the mean *SS*^*Rel*^ score of 0, we used a BP-funsim score of 0.4 as a cutoff to determine if an interacting protein shares F1 or F2 function. Consistent with Figure 6A and 6B, the majority of interacting proteins have F1 function for 18 out of 27 the moonlighting proteins (66.7%) (red bars). On the other hand, only nine moonlighting proteins (33.3%) have interacting proteins of F2 functions (blue bars), and among them interacting proteins with F2 function are dominant for three (11.1%) moonlighting proteins.

There are interacting proteins of moonlighting proteins that have functional similarity with both F1 and F2 functions of moonlighting proteins (shown by green bars in Figure 6C). Fifteen moonlighting proteins have in total of 30 interacting proteins with both F1 and F2 functions. We analyzed assigned GO terms of these interacting proteins by referring to literature and found that 18 out of 30 of these proteins are also moonlighting proteins while three proteins are multi-domain proteins. This result is summarized in Table 3. This result indicates that moonlighting proteins tend to interact with moonlighting proteins; thus, novel moonlighting proteins may be identified by analyzing PPIs of moonlighting proteins.

**Table 3:** **Interacting proteins that have both primary and secondary functions of moonlighting proteins in the MPR1-3 set**

**Moonlighting proteins**			**Interacting proteins**			
**Uniprot ID/Name** ^**a)**^	**Primary function** ^**b)**^	**Secondary function** ^**c)**^	**UniProt ID/Name** ^**d)**^	**Interacting protein function** ^**e)**^	**MP/non-MP** ^**f)**^	**Ref.**
P93834/HXK2	Glucose metabolism	Glucose signalling	Q42525/HXK1	1. Glycolysis	MP, I	[84-86]
				2. Sugar mediated signaling programmed cell death		
Q99798/ACO2	TCA cycle enzyme	Iron homeostasis	P21399/ACO1	1. Role in TCA cycle	MP, I	[87,88]
				mRNA binding and role in iron homeostasis		
P00924/ENO1	Galactose catabolism enzyme	Homotypic vacuole fusion	P00925/ENO2	1. Glycolysis	MP, I	[89]
				Vacuole fusion		
Q08438/Vhs3	Halotorance determinant	Coenzyme A biosynthesis	P36024/SIS2	1. CoA biosynthesis	MP, I	[90]
				Salt tolerance		
P28482/ERK2	MAP kinase	Transcriptional	P01100	1. Regulation of transcription	MP, II	[91,92]
		Repressor	/FOS	2. Activates phospholipid synthesis in growing cells (regulated by Mos/MAP kinase pathway)		
			Q15796/SMAD2-5	1. TGF signaling protein	Multi-domain	[93]
				2. Tumor suppressor, dual role in transcriptional activation		
			P05771/PRKCB	1. Serine/threonine-protein kinase, activates transcription.	MP, II	[94]
				Inhibition of the insulin gene transcription.		
			O43293	1. Serine/threonine kinase	MP, I	[95,96]
			/DAPK3	2. Role in apoptosis, transcription, regulation of cell polarity, contractile processes in non-muscle or smooth muscle cells, and cytokinesis		
			P14921/ETS1	Transcription factor	nonMP	-
			P19838/NFKB1	1. Transcription factor	MP, I	[97]
				2. Cytoplasmic retention of attached NF-kappa-B proteins by p105, generation of p50 by a co-translational processing, transcriptional repressor		
			O43318	1. MAPK	MP, II	[98,99]
			/MAP3K7	Regulates TF activator proteins		
Q99ML3/STAT3	Transcription factor	Electron transport chain	Q5EG47/Prkaa1-2	1. Protein kinase that phosphorylates TF	MP, II	[100]
				Regulation of cellular energy		
			Q62120/Jak2-3	1. Tyrosine protein kinase	Multi-domain	[101]
				Regulation of cellular signaling and cell cycle control		
			P05480/Src	1. Tyrosine protein kinase	Multi-domain	[102]
				Cytokine/cellular receptor		
O14786/Neuropilin-1	Vascular endothelial growth factor	Receptor for semaphorin III	Q14563/SEMA3A-G	1. Development of the olfactory system and in neuronal control of puberty	MP, II	[103]
				2. Ensures proper endothelial abundance of soluble and alternatively spliced form of VEGF receptor(flt1)		
			P15692/VEGFA	Vascular endothelial growth factor	nonMP	-
			P17948	1. VEGF receptor; plays negative role in angiogenesis in the embryo most likely by trapping VEGF	MP, I	[104]
			/FLT1/VEGFR/	2. Plays positive role in adulthood in a tyrosine kinase-dependent manner		
P54279/PMS2	Mismatch repair enzyme	Hypermutation of antibody variable chains	Q9JK91/Mlh1	1. Mismatch repair protein	MP, I	[105]
				Somatic hyper mutation		
P19971/PD-ECGF	Thymidine phosphorylase	Platelet-derived endothelial cell growth factor	P04183/TK1-2	1. Phosphotransferase activity	nonMP	-
			Q96B60	Deoxyribonucleotidase, mitochondrial	nonMP	-
			/NT5E, NT5M			
P06744/Neuroleukin	Phosphoglucose Isomerase	Differentiation, maturation mediator	P52789/HK2	1. Hexokinase-2	MP, III	[106]
				HK2 detachment causes apoptosis		
			P04075/ALDOA-C	1. Glycolysis and gluconeogenesis	MP, III	[107,108]
				Regulation of cell shape		
			P30613/PKLR	1. Pyruvate kinase	MP, III	[109]
				Mutation causes hemolytic anemia		
			P14618/PKM2	1. Pyruvate kinase	MP, III	[110]
				Programmed cell death		
P06795/P-glycoprotein	P-glycoprotein (transporter)	Regulator of cell-swelling ion channel (K+/Cl-)	P41233/Abca1	Anion transporter	nonMP	-
Q91XR9/Phospholipid hydroperoxide glutathione peroxidase	Antioxidant of mature sperm	Structural protein of the mitochondrial capsule	Q60928/Ggt1	1. Part of the cell antioxidant defense mechanism	MP, IV	[111,112]
				2. Indirectly regulates multiple aspects of skeletal biology		
Q9P2J5/Leucine-tRNA ligase	tRNA synthetases	Translocation and activation of mTORC1 to lysosomal membrane	Q9H6Q3/MARS	Methionine-tRNA ligase, cytoplasmic	nonMP	-
			Q6P0M4/IARS	tRNA aminoacylation for protein translation	nonMP	-
P11325/Nam2p	Mitochondrial leucyl-tRNA synthetase	bI4 mitochondrial RNA splicing activity	P26637/CDC60	Leucine-tRNA ligase, cytoplasmic	nonMP	-
P19414/ACO1	TCA cycle enzyme	Mitochondrial DNA stability	P33421/SDH3	Succinate dehydrogenase involved in mt-electron transport chain	nonMP	-

This table corresponds to Figure 6C.

^a)^The name and UniProt ID of the moonlighting proteins in the MPR1-3 set.

^b)^Primary function and ^c)^ secondary function of the moonlighting protein.

^d)^The name and the UniProt ID of interacting proteins of the moonlighting protein shown in the left column.

^e)^Multiple functions (if any) of the interacting protein.

^f)^This column indicates if the interacting protein is a moonlighting protein (MP), not (non-MP), or multi-domain multi-functional protein (Multi-domain). The roman numerals, I to III, indicate the category of moonlighting proteins (see Table 1 caption).

We discuss two such cases. The first example is mismatch repair endonuclease PMS2 (P54279) in mouse, which also contributes to somatic hypermutation [113]. It has just one interacting protein, which is another DNA mismatch repair protein Mlh1 (Q9JK91) that is also involved in somatic hypermutation [105]. Thus, this is an example of two interacting moonlighting proteins that have the same primary and secondary functions.

The second example is mitogen activated protein kinase 1 (ERK2) (P28482) in human. This protein is MAP kinase and moonlights as a transcriptional repressor [114]. It has 187 interacting proteins in the PPI network, among which there are ten proteins with both F1 and F2 functions. One of the interacting partners is death-associated protein kinase 3 (DAPK3, UniProt: O43293), which enhances transcriptional activities of STAT3/P40763 by phosphorylating them. Besides the kinase function, DARPK3 is known to have multiple secondary functions, including involvement in apoptosis [39], roles in transcription (same as the secondary function of ERK2), regulation of cell polarity, contractile processes in non-muscle or smooth muscle cells, and cytokinesis [40]. Thus, in this example, among interacting moonlighting proteins that share both F1 and F2 functions, one of them has more secondary functions.

### Co-expressed proteins

**Figure 7:**
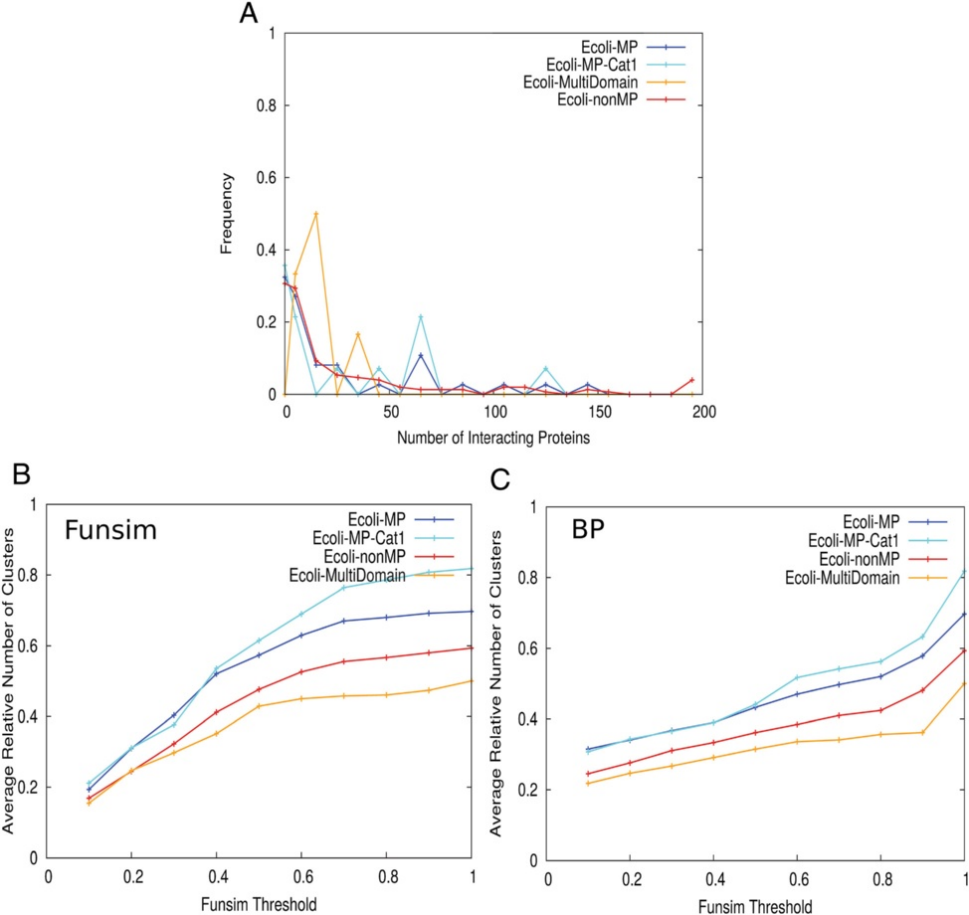
**Gene expression profile analysis.** Average number of clusters of interacting proteins relative to the number of proteins interacting by gene expression. Proteins considered to be interacting are the top 2% of proteins in the Gene Expression network of *E. coli* sorted in terms of the Pearson correlation coefficient. **(A)** Histogram of number of interacting proteins. **(B)** Functional clustering using Funsim (BP, MF, CC) score thresholds between 0.1 and 1.0. **(C)** Functional clustering using Funsim (BP) score thresholds between 0.1 and 1.0.

Next, we investigated functions of co-expressed genes with moonlighting proteins in *E. coli*. The *E. coli* gene expression data were taken from the COLOMBOS database [115], which contains expression data of 4295 genes in 2369 contrasts. We calculated the Pearson correlation coefficient of expression levels of each pair of genes and selected pairs as co-expressed if the absolute value of the correlation coefficient is ranked within the top 2% largest values among all the pairs. The number of co-expressed genes of moonlighting and non-moonlighting proteins do not have large difference, except for a peak observed at 65 for the moonlighting proteins (Figure 7A), which consists of four moonlighting proteins (P77489, P0A8Q3, P0AC47, and P25516). Then, similar to the analysis in Figure 5B and 5C, we computed functional clustering profile for co-expressed genes of *E. coli* moonlighting proteins to see if co-expressed genes have functional divergence. The clustering profile using the funsim score (Figure 7B) and the BP-funsim score (Figure 7C) showed that the moonlighting proteins have a slightly larger average number of clusters of functionally similar proteins per co-expressed genes than that for non-moonlighting proteins, although this difference is not statistically significant (Additional file 1: Table S1). The same conclusion was obtained when we defined co-expressed genes as those which have over 0.4 of the correlation coefficient value (data not shown).

### Phylogenetically related genes

**Figure 8:**
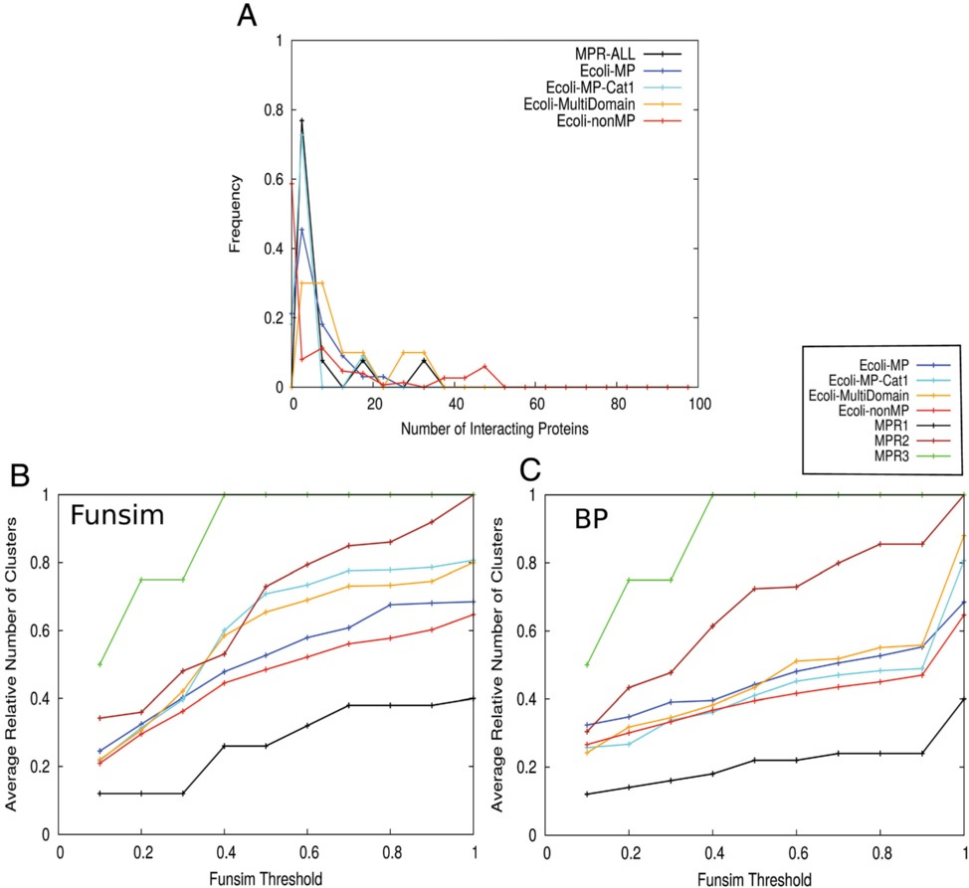
**Phylogenetic profile analysis.** Average number of clusters of phylogenetically related proteins relative to the number of phylogenetically related proteins. Phylogenetically related proteins are taken from the STRING database. **(A)** The histogram of number of phylogenetically related proteins. **(B)** Functional clustering using Funsim (BP, MF, CC) score with thresholds between 0.1 and 1.0. **(C)** Functional clustering using Funsim (BP) score with thresholds from 0.1 to 1.0.

We further analyzed genes that have similar comparative genomic context to the moonlighting proteins [41]. Using the STRING database, for a protein of interest, we selected proteins as phylogenetically related if they were located in the neighbourhood of the target genes, were found to co-occur or co-absent, or were fused in multiple genomes. Concretely, genes that have a sufficient score (> 0.7 as recommended by STRING) at “neighborhood”, “co-occurrence”, or “gene-fusion” in the STRING database [83] were selected. It has been observed that phylogenetically co-related proteins are functionally related in many cases [41]. Figure 8 shows the clustering profiles of phylogenetically related proteins of the moonlighting and non-moonlighting proteins.

A larger fraction of the non-moonlighting proteins have no phylogenetically related proteins as compared with the moonlighting ones (0 at the x-axis in Figure 8A). The clustering profiles using the funsim score (Figure 8B) and the BP-funsim score (Figure 8C) show that the *E. coli* moonlighting proteins have slightly more functional clusters on average, i.e. more functional divergence in their phylogenetically related proteins, than their non-moonlighting counterparts. The p-value of this difference in the number of functional clusters was 0.08 at the score threshold of 0.8 in the funsim score (Figure 8B) and larger than 0.05 for the BP-funsim score profile (Figure 8C). Comparing with the MPR1-3 sets, on average MPR2 and MPR3 have a higher number of clusters than the *E. coli* moonlighting and non-moonlighting proteins, while the MPR1 set has less functional divergence in their phylogenetically related proteins.

### Genetic interaction network analysis

**Figure 9:**
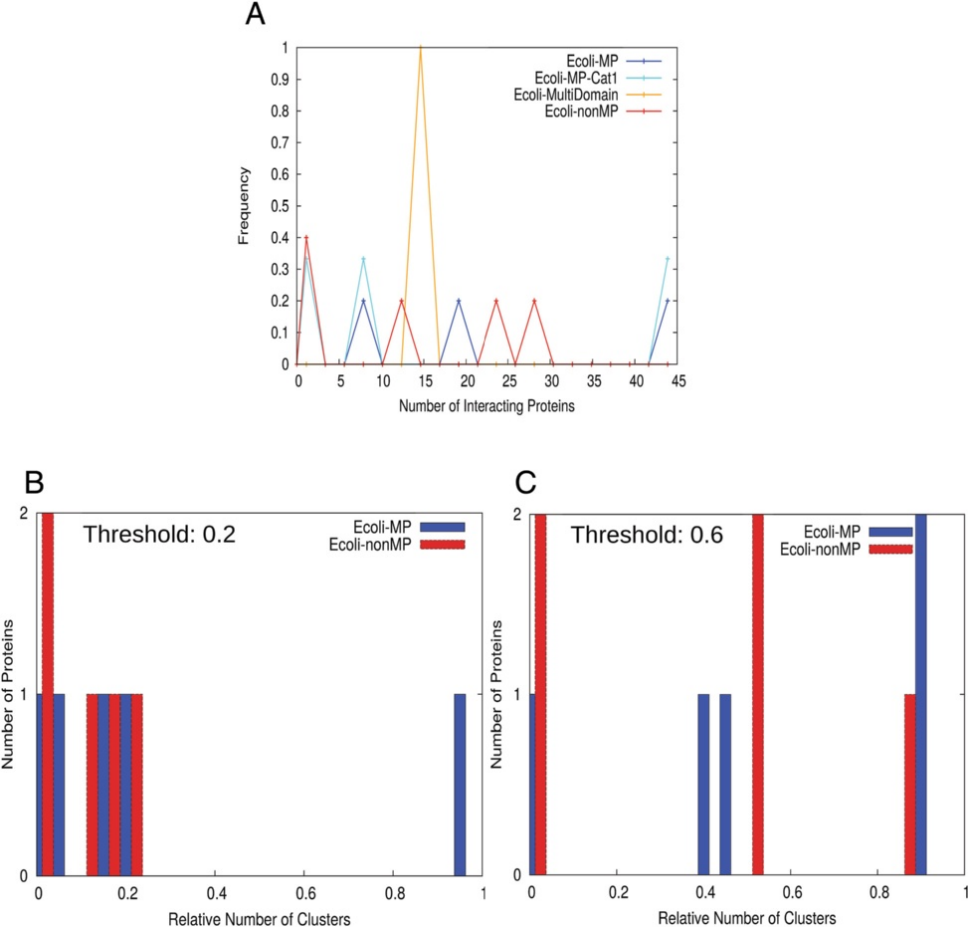
**Genetic interaction network analysis.** The number of interacting proteins in the genetic interaction network of *E. coli*. **(A)** The number of interacting proteins selected with a Pearson correlation cutoff of 0.2. *E. coli* MP and non-MP, multi-domain multi-functional proteins, and the first category *E. coli* MPs are plotted. **(B)** The number of clusters of interacting proteins for individual *E. coli* moonlighting (blue) and non-moonlighting (red) proteins at BP-funsim threshold of 0.2. **(C)** The number of clusters of interacting proteins for individual *E. coli* moonlighting (blue) and non-moonlighting (red) proteins at BP-funsim threshold of 0.6.

The last omics data we analyzed were genetic interactions. A genetically interacting gene pair was identified by examining the growth curves of a single gene knockout mutant and a double gene knockout mutant. In general, genes in the same pathway tend to show positive interaction and those in parallel pathways show negative or synthetic lethality [116]. Genetic interactions in *E. coli* were identified by Takeuchi et al. [117] using conjugation methods reported as GIANT-coli [118] and eSGA [119] with an improved quantitative measurement [120]. This dataset includes genetic interaction data for 215 genes against 3868 genes, which results in total of 813,560 gene combinations. Among them, 2009 pairs were identified as genetically interacting, which were defined as those have a correlation coefficient of over 0.2 in the maximum growth rate in time-series measurements [117]. The interacting gene pairs overlap with a small portion of the *E. coli* moonlighting and non-moonlighting proteins: 5 out of 33 moonlighting proteins, 3 out of 16 first category moonlighting proteins, and 5 out of 150 non-moonlighting proteins. Using these shared proteins, we performed the clustering profile analysis (Figure 9).

Moonlighting and non-moonlighting proteins do not seem to have difference in the number of genetic interactions (Figure 9A) and the number of functional clusters (Figure 9B & 9C), although the number of proteins available for the analysis was too small to make a firm conclusion. In terms of the number of genetic interactions (Figure 9A), there is one moonlighting protein that has 43 genetic interactions. This protein is a subunit of fumarate reductose flavoprotein in *E. coli* (P00363), which we classified as a first category moonlighting protein (Table 1). The 43 interacting proteins belong to 30 different pathways. Panels B & C in Figure 9 show histograms of the number of functional clusters of genetically interacting proteins for the *E. coli* moonlighting and non-moonlighting proteins at the BP-funsim thresholds of 0.2 and 0.6. There is a moonlighting protein that interacts with two proteins with very different functions (the bar at × = 1.0 in Figure 9B). This protein is P23895, a third category/weak moonlighting protein identified to function as a multidrug transporter and in DNA damage response. It interacts with P77368 (UPF0098 family protein inferred by homology) and P75719 (endopeptidase that performs host cell lysis).

To summarize the omics data analyses, we observed a clear tendency for moonlighting proteins to have physical interactions with more diverse classes of proteins and most of these proteins share the primary function of the moonlighting protein with which they interact. Moreover, it was found that moonlighting proteins frequently physically interact with other moonlighting proteins. In terms of gene expression and phylogenetically related proteins, a weak trend was observed that on average moonlighting proteins interact with more functionally diverse proteins, although not all of the cases were statistically significant.

### Structural properties of moonlighting proteins

Now we turn our attention to structural properties of moonlighting proteins, namely intrinsically disordered regions and ligand binding sites. An intrinsically disordered region in a protein lacks a well-defined tertiary structure in its native condition. Intrinsically disordered regions have been found to have important roles in protein function [121], often serving as binding sites for proteins. There are moonlighting proteins that can both activate and inhibit their binding partners in the same or overlapping binding regions which have been found to be disordered. These proteins can bind the same partner in different conformations or bind to completely different partners through the disordered binding regions [122]. Here, we examined the prevalence of disordered regions in the proteins in MPR1-3 and the *E. coli* moonlighting and non-moonlighting proteins. Disordered regions in the proteins were obtained from the D2P2 database [123].

**Figure 10:**
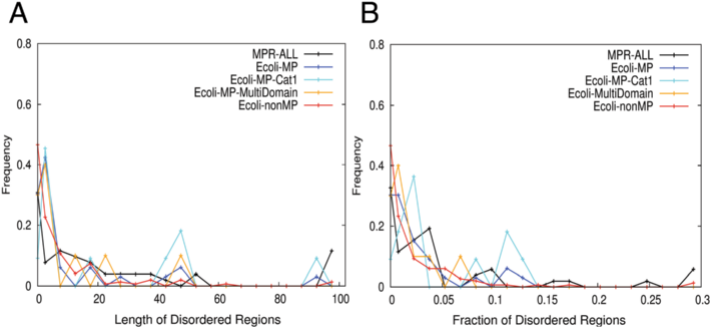
**Disordered region of moonlighting and non-moonlighting proteins.** Histograms of the disordered regions in moonlighting and non-moonlighting proteins. Five datasets are plotted: MPR1-3 (MPR-All), *E. coli* moonlighting proteins (Ecoli-MP), *E. coli* moonlighting proteins in the first category (Ecoli-MP-Cat1), multi-domain multi-functional proteins, and *E. coli* non-moonlighting proteins (Ecoli-nonMP). **(A)** Length of the disordered regions; **(B)** Fraction of the length of disordered regions relative to the whole sequence length of the proteins.

The total length of disordered regions and their fraction relative to the full length of a protein are shown in Figure 10. The distributions for moonlighting proteins and non-moonlighting proteins were overall similar, both having the peak at lower end within disordered region lengths 0 to 5. However, it is noteworthy that moonlighting proteins had a smaller fraction of proteins with no disordered regions (Figure 10A) and more moonlighting proteins had a larger fraction of disordered regions (Figure 10B). Moonlighting proteins had a small peak for disordered regions of 47 residues in length and slightly higher frequency for disordered regions of over 90 residues (Figure 10A). The peak of the moonlighting proteins at 47 residue-long disordered regions (Figure 10A) consists of four proteins, fumarate reductase (P00363), ribonuclease R (P21499) deferrochelatase (P31545), and GTPase ObgE (P42641). Moonlighting proteins with a large fraction of disordered region include anion exchange protein 3 (P48751) and phosphopantothenoylcysteine decarboxylase subunit VHS3 (Q08438) and subunit S1S2 (P36024). Anion exchange protein 3 does not have known physical interactions with other proteins while the two subunits of phosphopantothenoylcysteine decarboxylase have eight physical interactions in the PPI network.

### Ligand binding sites

**Figure 11:**
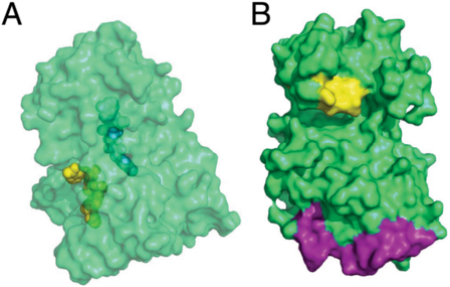
**Moonlighting protein structures.** Tertiary structures of moonlighting proteins. **(A)** human dihydrolipoamide dehydrogenase (PDB ID: 1ZMC-A). It binds NAD shown in yellow at residues 208, 243, 279 (“NAD binding” classified as both F1 and F2 function) and FAD shown in cyan at residues 54, 119, 320 (“FAD binding” classified as F2 term). **(B)** mitogen activated protein kinase 1 (PDB ID: 4G6N). It binds ATP (related to F1 function) at residues 31–39 and 54 (shown in yellow), and DNA (related to F2 function) with residues 259–277 (purple).

Finally, we discuss ligand binding sites in the tertiary structures of moonlighting proteins that are related to either of their primary or secondary functions. Such examples are limited since the tertiary structures of the proteins must be available for the analysis and multiple bound ligands need to be involved in the functions. Sixteen proteins in the MPR1-3 sets have their tertiary structures available in PDB [124,125]. Among them, we found six structures that have two ligands that bind to physically different locations. We discuss two cases below, because the other four are multi-domain proteins (Figure 11). These two proteins to be discussed are one-domain proteins according to Pfam.

The first example is dihydrolipoamide dehydrogenease (DLD) in human (P09622) (Figure 11A). The primary function of this protein is as a mitochondrial enzyme in energy metabolism and its secondary function is protease. To perform the primary function, it utilizes dihydrolipoic acid and NAD+ to generate lipoic acid. Experiments suggest that mutations that destabilize a DLD homodimer can simultaneously induce the loss of a primary metabolic activity and the gain of a moonlighting proteolytic activity [59]. It was also pointed out that the moonlighting proteolytic activity of DLD could arise under pathological conditions, including the presence of dimer-destabilizing mutations or the acidification of the mitochondrial matrix. The latter condition disrupts the quaternary structure of DLD, leading to a decrease in the dehydrogenase activity and increase in the diaphorase activity, which is a FAD and NAD dependent activity. Based on these information we classified “NAD (nicotinamide adenine dinucleotide) or NADH binding” (GO:0051287) to both functions and term “FAD (flavin adenine dicucleotide) or FADH2 binding” (GO:0050660) to the secondary function. A crystal structure of DLD (PDB ID: 1ZMC-A) shows that the NAD and FAD binding sites are located in physically separate regions in the protein surface.

The second example is MAP kinase (ERK2) in human. The secondary function of this protein was identified as a DNA binding transcriptional repressor that regulates interferon gamma signalling [64]. Naturally, binding ATP is related to the primary function as a kinase (GO:0005524) while “DNA binding” (GO:0003677) belongs to the secondary function. As shown in Figure 11B, the binding sites for ATP and DNA are located quite far apart in the protein structure.

To summarize the structural analyses, about 48% of moonlighting proteins have disordered regions longer than five residues and this percentage is larger than that of non-moonlighting ones (29%). Also examples are observed in which moonlighting proteins have relatively longer disordered regions. In terms of the tertiary structures, examples are found where ligand (including DNA) binding sites that are related to either the primary or secondary functions are located in distinct regions on the protein surface. These structural features may be useful for predicting the existence of secondary function of proteins when combined with other evidences.

## Discussion

Moonlighting proteins have more than one independent function. It is speculated that moonlighting proteins are not few in number and expected to be found more in the future. Identification of moonlighting proteins indicates that potential secondary functions need to be considered when it comes to protein function, which has significant impact on functional genomics, proteomics, and computational gene function annotation [10].

In the first part of this work, we examined current GO annotations of known moonlighting proteins. We found that the GO term annotations for moonlighting proteins can be clustered into more than one cluster based on the semantic similarity between pairs of GO terms. Thus, even in the case that moonlighting proteins are not labelled as such in the annotation database, we will be able to identify them by observing the functional divergence of annotated GO terms. Based on this intuitive observation, we analyzed *E. coli* proteins in the database and identified novel moonlighting proteins.

The second half of this work addressed characteristics of moonlighting proteins in omics data and their tertiary structures. We found that moonlighting proteins tend to physically interact with proteins of diverse functions. The same trend, although weak, was observed for proteins that are co-expressed with or are phylogenetically related to moonlighting proteins. The majority of interacting proteins of a moonlighting protein shared the primary function of the moonlighting protein and we found that a substantial fraction of the interacting proteins were themselves moonlighting proteins.

The characteristics of moonlighting proteins were investigated by comparing their features with those of non-moonlighting proteins. In general, finding examples that do not possess a certain property is not straightforward as future research may find that the examples actually do have the property. So are non-moonlighting proteins – there is an undeniable possibility that non-moonlighting proteins used in this study will be found as moonlighting in the future. Nevertheless we believe the current research is valuable and has contributed in progressing our understanding of moonlighting proteins because the non-moonlighting proteins were selected in a reasonable way and also because the differences and similarities of characteristics of moonlighting and non-moonlighting proteins were clarified that can serve as hypotheses in the future works. We would also like to point out that similar approaches of selecting negative data sets were taken in analyzing protein-protein interactions (by constructing a non-interacting protein dataset, Negatome [126]) and in analyzing proteins with particular functions (by constructing the NoGo database [127]), which contributed in development of computational prediction methods and thereby advance our understanding and the research field.

As for the structural aspects, a larger fraction of moonlighting proteins than non-moonlighting ones had intrinsically disordered regions. We have also discussed examples that ligands related to the primary and secondary functions bind at distinct regions in the tertiary structure. Application of structural analyses is limited because obviously protein structure information is needed. However, we would like to point out that disordered regions can be well predicted from a protein sequence and ligand binding sites can be also predicted in an experimentally determined protein structure or in a computational structure model.

We observed significant functional divergence in physically interacting proteins with moonlighting proteins, which could be a good feature to use for predicting of moonlighting proteins. However, the other features of moonlighting proteins in omics data were weak. Thus, predicting moonlighting proteins from an individual feature may not be an easy task. This also reminds us that moonlighting functions are observed in various physiological conditions of a cell, which differ for each moonlighting protein. Therefore, ultimately, prediction of moonlighting proteins or secondary functions of a protein needs a holistic understanding of behavior of molecules in a cell. In practice, this means that integrating various different cell-level data will be effective in prediction, which includes proteomics, ionomics, phenotypic data of mutants, bioinformatics predictions, computational simulations of pathways, and molecular dynamics of biomolecules. Such an automated computational method would be useful in resolving many ambiguities in proteomics analysis as well as in unfolding many complexities of protein functions. Improved understanding of moonlighting functions of proteins can be a touchstone for our knowledge of molecular biology, because it requires comprehensive, multilevel data and deep knowledge of the cell.

## Conclusions

The functional diversity of moonlighting proteins poses a challenge to their experimental identification as well as computational annotation [10,29]. Our method enables identification of novel moonlighting proteins from a current database, even when they are not explicitly annotated as such. Moreover, we showed that potential moonlighting proteins without sufficient functional annotations could be identified by considering available omics-scale data and computational structural predictions. Our findings open up a new opportunity to investigate the multi-functional nature of proteins at a systems level and explore the complex functional interplay of proteins in a cell.

## Methods

### Dataset of known moonlighting proteins

We constructed three datasets of experimentally confirmed moonlighting proteins from two review articles [1,18] and papers we collected from the PubMed database. They are called the MPR1 (24) [18], MPR2 (18) [1], and MPR3 (16) set, respectively. In the parentheses is the number of moonlighting proteins in the each dataset. The MPR1 dataset was used in our previous study [29]. The three datasets are available at http://kiharalab.org/MoonlightingDatasets. The list of proteins in the MPR3 set is provided in Table 4. In MPR1 and MPR2, we found four proteins (ATF2, PutA, neuropilin-I, and BirA) are multi-domain proteins. Although these four proteins are also listed as moonlighting proteins in MultitaskProtDB and MoonProt, we excluded them from the dataset in all the results except for the bar graphs in Figure 2 and Figure 6 where these proteins are noted with asterisk (*). For each of the moonlighting proteins in the three datasets, GO term annotations in UniProt were classified into four classes by referring to textual description of the protein’s function in literature: GO annotations that described the “primary” function of the protein (Function 1, F1), GO annotations that describe “secondary” function (Function 2, F2), GO annotations that correspond to both functions of the protein (usually general GO terms at a higher depth of the GO hierarchy), and lastly, GO annotations whose functional association to either of the two functions were unclear. In cases that the description of the secondary function of a moonlighting protein was absent or incomplete in UniProt, we annotated the protein with appropriate GO terms selected from the GO database.

**Table 4:** **The MPR3 moonlighting protein dataset**

**Uniprot ID/Protein name**	**Organism**	**Primary function**	**Secondary function(s)**	**Ref**
P79149/Pinin	Canis familiaris	Induce junction formation and enhance cell aggregation	Component of the RNP structure	[128]
P27487/DPP4	Homo sapiens	Serine protease	1. Cell surface glycoprotein receptor for CAV1	[129]
			2. Co-stimulatory protein involving in T-cell receptor-mediated T-cell activation and proliferation.	
			3. Binding collagen and fibronectin	
			4. Involvement in apoptosis	
Q91XR9/GPx-4	Mus musculus	Antioxidant of mature sperm	Structural protein of the mitochondrial capsule	[130]
O35242/FAN	Mus musculus	Apoptosis	Inflammatory signalling	[131]
E3D2R2/Fructose-1, 6-bisphosphate aldolase	Neisseria meningitidis	Glycolytic enzyme	Host-cell invasion	[132]
Q7L0Y3/MRP1	Homo sapiens	tRNA methyltransferase	Dehydrogenase	[133]
Q9Y7F0/Peroxiredoxin TSA1	Candida albicans	Antioxidant against sulfur-containing radicals	Involved in morphology	[134]
P48237/CCM1	Saccharomyces cerevisiae	Introns removal in mRNA maturation	Maintains the steady-state levels of the mitoribosome small subunit RNA	[135]
P11325/Nam2p	Saccharomyces cerevisiae	Mitochondrial leucyl-tRNA synthetase	Mitochondrial RNA splicing activity	[136]
Q9P2J5/LeuRS	Homo sapiens	tRNA synthetase	Translocation and activation of mTORC1 to lysosomal membrane	[137]
P47897/GlnRS	Homo sapiens	tRNA synthetase	Suppresses apoptotic acitivities	[137]
Q6DRC0/SerRS	Danio rerio	tRNA synthetase	Regulates development of closed circulatory system	[137-139]
P00883/Fructose-bisphosphate aldolase A	Oryctolagus cuniculus	Glycolytic enzyme	Regulation of cell mobility	[140]
P0A518/Cpn60-1	Mycobacterium tuberculosis	Prototypic molecular chaperone	Osteoclast-inhibitory action	[141]
P0A518/Cpn60-2	Mycobacterium tuberculosis	Prototypic molecular chaperone	Stimulates macrophage pro-inflammatory cytokine synthesis	[141]

### Semantic similarity of GO term pair and funsim score

We used the relevance semantic similarity score (*SS*^*Rel*^) [142] for computing functional similarity of a pair of GO terms, c_1_ and c_2_:

1 $$ S{S}^{\mathrm{Re}l}\left({c}_1,{c}_2\right){=}_{c\in S\left({c}_1,{c}_2\right)}^{\max}\left(\frac{2 \log\;p(c)}{ \log\;p\left({c}_1\right)+ \log\;p\left({c}_2\right)}\left(1-p(c)\right)\right) $$

Here *p(c)* is the probability of a GO term *c*, which is defined as the fraction of the occurrence of *c* in the GO Database [35,36]. The root of the ontology has a probability of 1.0. *s(c*_*1*_*,c*_*2*_*)* is the set of common ancestors of the GO terms *c*_*1*_ and *c*_*2*_*.* The first term considers the relative depth of the common ancestor *c* to the depth of the two terms *c*_*1*_ and *c*_*2*_ while the second term takes into account how rare it is to identify the common ancestor *c* by chance.

To quantify the functional similarity of two proteins, both of which are annotated with a set of GO terms, we used the funsim score [31]. The funsim score of two sets of terms, *GO*^*A*^ and *GO*^*B*^ of respective size of *N* and *M*, is calculated from an all-by-all similarity matrix *s*_*ij*_.

2 $$ {S}_{ij}=sim{\left(G{O}_i^A,G{O}_j^B\right)}_{\forall i\in \left\{1..N\right\},\forall j\in \left\{1..M\right\}} $$

*sim(GO*_*i*_^*A*^*, GO*_*i*_^*B*^*)* is the relevance similarity score for *GO*_*i*_^*A*^ and *GO*_*j*_^*B*^. Since the relevance similarity score is defined only for GO pairs of the same category, a matrix is computed separately for the three categories, Biological Process (BP), Molecular Function (MF), and Cellular Component (CC). Then, the GOscore of the matrix of each GO category is computed as follows:

3 $$ G{O}_{score}= \max \left(\frac{1}{N}{\displaystyle {\sum_{i=1}^N}_{1\le i\le M}^{\max\;{S}_{ij},}\frac{1}{M}}{\displaystyle {\sum_{i=1}^M}_{1\le i\le N}^{\max\;{S}_{ij}}}\right) $$

GOscore will be any of the three category scores (MFscore, BPscore, CCscore). Finally the funsim score is computed as

4 $$ funsim=\frac{1}{3}\left[{\left(\frac{MFscore}{ \max (MFscore)}\right)}^2+{\left(\frac{BPscore}{ \max (BPscore)}\right)}^2+{\left(\frac{CCscore}{ \max (CCscore)}\right)}^2\right], $$

where *max(GOscore)* = 1 (maximum possible GOscore) and the range of the funSim score is (0,1).

## Reviewers’ comments

### Reviewer 1 (First Round): Dr. Michael Galperin (National Center for Biotechnology Information, National Library of Medicine, National Institutes of Health, USA)

General comment:

This manuscript addresses an intriguing problem of multi-functionality in proteins. "Moonlighting" proteins that have two or more distinct functions are being discovered at a steady pace which makes this contribution important and timely. Having said that the current version of the manuscript has a number of problems that need to be fixed before it can be considered for publication.

1. This manuscript inexplicably ignores the existence a publicly available database of moonlighting proteins MultitaskProtDB (http://wallace.uab.es/multitask described in Hernández et al. Nucleic Acids Res. 42517-D520 2014). There is also MoonProt (http://www.moonlightingproteins.org/ Mani et al. MS thesis University of Illinois at Chicago 2014). A careful comparison of the results of this study with the data presented in those two databases is essential to this work.

Authors’ response: *We searched the 43 moonlighting (Table**1**) and multi-functional multi-domain proteins (Table **2**) we identified from E. coli against both MultitaskProtDB and MoonProt. In MultitaskProtDB we found one protein (b0161/P0C0V0/DegP) in the 43 proteins. The 43 proteins we identified from E. coli include aconitases (AcnA and AcnB), which are not included in MultitaskProtDB but their homologs in three species (aconitase in H. Sapiens, M. Tuberculosis, S. Cerevisiae) are included. In MoonProt, we found 3 proteins (b0161/P0C0V0/DegP, b4260/P68767/pepA, and b4390/P27278/nadR). nadR was found in Table**2**, which is for multi-functional multi-domain proteins. This database, too, contains aconitases of four organisms (H. Sapiens, M. Tuberculosis, S. Cerevisiae, and B. Taurus) but not one from E. coli. Thus, out of 33 new moonlighting proteins listed in Table**1**, only two are found in the existing two databases.*

*We have indicated the three proteins in Table**1**and Table **2** that are found in MultitaskProtDB and MoonProt with † (dagger) and mentioned in the text as follows: “The identified 33 moonlighting proteins (Table**1**) and 10 multi-domain multi-function proteins (Table**2**) do not have many overlap with the MoonProt database and MultitaskProtDB. Only two (PepA and DegP) with Table**1**and one (NadR) in Table **2**” (page 13).*

2. This work fails to distinguish between truly moonlighting proteins where each part of the polypeptide chain participates in two different activities and multi-domain proteins that combine in a single polypeptide chain two or more different domains each with its own specific function. The authors correctly define moonlighting as not "not a consequence of gene fusions" (p.3 l.7) but include in the manuscript numerous examples of proteins that have acquired different functions as a result of fusion of two or more genes encoding distinct domains. For E. coli examples of two-domain proteins listed in Table 1 include ThrA (b0002) CysG (b3368) MetL (b3940) NadR (b4390) HldE (b3052) SpoT (b3650) to name just a few. In addition ATF2 PutA neuropilin-I and BirA which are discussed on pp. 23-24 and displayed on Figure 11 C-G are also multi-domain proteins. As correctly stated by the authors their distinct functions reside in distinct domains and therefore none of these proteins is truly moonlighting.

Authors’ response: *We appreciate this important comment by the reviewer. In response to this comment, we have consulted with the Pfam database to find domains in the 43 E. coli proteins that were originally listed in Table**1**. Then, to determine if the two functions (primary and secondary functions) of the proteins originate from different domains, we analyzed GO terms associated with each Pfam domain. In case the GO terms associated with a domain are too general or incomplete, we have also examined the domain’s text description in the Pfam database. As a result, we identified ten proteins as multi-domain proteins whose multiple functions are caused by different domains. These proteins include all the six proteins pointed out by the reviewer, ThrA (b0002), CysG (b3368), MetL (b3940), NadR (b4390), HldE (b3052), and SpoT (b3650) and four more proteins. (NadR was found in the MoonProt database, too). We excluded these ten multi-domain proteins from Table**1**and separately listed them in a new table, Table**2**. Initially, in Table**1**, there were five proteins that were categorized as category III: Multi reaction enzymes. However, since all the five multi-reaction enzymes turned out to be multi-domain proteins, now the category III is removed from Table**1*.

*Moreover, we have removed these ten multi-domain proteins from the E.coli moonlighting proteins datasets, Ecoli-MP and Ecoli-MP-Cat1 (the First Category moonlighting proteins in E.coli), and redone all the subsequent analyses (Figures**3**and**10**). The ten multi-domain proteins were separately plotted in the Figures. Statistical analyses, namely, p-values in Supplementary Table S1 and the Friedman test for Figure**3**were also recomputed with the revised datasets. Importantly, removing the 10 proteins from the analyses did not change the overall trends and conclusions.*

*The four proteins in Figure**11**pointed out by reviewer (ATF2, PutA, neuropilin-I, and BirA) were also confirmed as multi-domain proteins with multiple functions by consulting with the Pfam database. Although all of them are included in both moonlighting protein databases - MultitaskProtDB and MoonProt, we excluded them from Figure**11**. But we kept them in the bar graphs in Figures**2**and**7*and marked them with asterisk *.

Specific comments.

p. 5 l. 14. "poses a challenge to the fundamental concept that genotype can explain phenotype" Please remove or at least reformulate. Genotype cannot "explain" anything only a human can. I do not see how moonlighting proteins could challenge the fundamental concept that genotype defines phenotype. Mutations in many genes have pleiotropic phenotypes even without any moonlighting.

Authors’ response: *We deleted the whole sentence.*

p. 6 l. 14. “the number of currently confirmed moonlighting proteins is too small”. Just how many such proteins are there? Have you examined the existing databases of such proteins MultitaskProtDB and MoonProt (see above)?

Authors’ response: *MultitaskProtDB and MoonProt have 288 and 289 entries, respectively. We rephrased the sentence as follows: “systematic studies of moonlighting proteins are still in their early stage for obtaining a comprehensive picture of proteins’ moonlighting functions and also to develop computational methods for predicting moonlighting proteins.”*

Ref. 19 cites the 1990 BLAST paper which described the first ungapped version of BLAST program. Did you actually use the ungapped version (which is quite difficult to find these days)? If not you should cite the 1997 BLAST paper (ref. 29) or the later ones.

Authors’ response: *We cited the 1997 version of the paper as pointed out.*

The references to the descriptions of Pfam (ref. 22) InterPro (ref. 23) GO (ref. 34) STRING (ref. 43) COLOMBOS (ref. 47) and PDB (ref. 56) are all outdated. If you used recent versions of these databases you should cite their most recent descriptions as recommended on the respective web sites.Authors’ response: *For Pfam, InterPro, STRING, and COLOMBOS, we now cited papers that were published in 2014, 2011, 2014, and 2014, respectively. The paper we originally cited for GO and PDB were those recommended on their respective websites (GO:*http://geneontology.org/page/go-citation-policy, *PDB:*http://www.rcsb.org/pdb/static.do?p=general_information/about_pdb/policies_references.html#References*). However, as suggested we now added 2013 papers for these two databases.*

Table 1 is poorly prepared and must be carefully revised.

- Protein name should start from a capital letter.

Authors’ response: *We corrected them.*

- There must be some order in the list (e.g. by gene name or b-number)

Authors’ response: *The list was sorted first by the category of moonlighting proteins (I to III), then further sorted according to b-number of proteins.*

- Why AcnB is (correctly) annotated as? Aconitate hydratase? but AcnA is only annotated as a? TCA cycle enzyme??

Authors’ response*: Now both annotated as “Aconitate hydratase”.*

- CheA is a Chemotaxis sensor kinase not "Chematoxis"

Authors’ response: *We changed it to “Chemotaxis sensor kinase” as pointed out.*

- While ObgA might indeed have multiple functions 'GTPase' and 'Chromosome segregation' is the same function.

Authors’ response: *The second function description changed to “Role in ribosome biogenesis”*

Quality of written English: Needs some language corrections before being published

Authors’ response: *We made language corrections with help by a native English speaker.*

### Reviewer 1 (Second Round): Dr. Michael Galperin

The revised manuscript looks fine to me but several minor corrections need to be made (these comments do not need to be included in the printed version):

Abstract

Background, 1st sentence: change “more than one cellular function” to “two or more cellular functions” (functions need to be plural to correspond to the subsequent “which are”). Authors’ response: *We corrected it to “two or more cellular functions”.*

Results, 5th sentence: “most of the physically interacting proteins share the primary function of the interacting moonlighting proteins” - could you explain that in simpler terms?

Authors’ response: *We rephrased it to “most of the physically interacting partners of moonlighting proteins share the latter’s primary functions”.*

Conclusion, 2nd sentence: change “function annotations in a database” to “functional annotations in public databases”

Authors’ response: *We changed the phrase as suggested.*

Main text

Background, 3d sentence: remove “first” from “first by Jeffrey [1]”. There have been two earlier papers, PMID: 8543908, 9663383.

Authors’ response: *Thank you for pointing out the two papers, which we missed. In addition to modifying the phrase as suggested we also cited the two papers.*

Quality of written English: Acceptable

### Reviewer 2 (First Round): Dr. Eugine Koonin (National Center for Biotechnology Information, National Library of Medicine, National Institutes of Health, USA)

Khan et al. report an extensive computational analysis of "moonlighting proteins". They correctly note that bioinformatic study of such proteins presents a difficult challenge. Much to my regret, I do not actually believe that the present manuscript meets the challenge to enhance the existing understanding of the moonlighting phenomenon. Moonlighting is not easy to define, it is one of the situations that are rather typical in biology where the "classic" examples are clear and compelling (see crystallins) but few and far between whereas away from the spotlight, matters become fuzzy. I am actually inclined to think that all proteins perform multiple roles in organisms and are at some level moonlighting. The reasons why we think of some but not other proteins as moonlighting have to do mostly with the level of our knowledge and the feasibility of defining discrete functional roles for a given protein. Consequently, I am deeply skeptical about the validity of the control set of "non-moonlighting" proteins and about any comparative analyses that attempt to contrast properties of moonlighting and non-moonlighting proteins.

Authors’ response: *We understand the reviewer’s concern about the validity of the non-moonlighting proteins. We agree that the selection of non-moonlighting proteins is based on our current knowledge of function of the proteins and it is possible that secondary function may be found for the proteins in the future. However, to understand characteristics of a certain group of proteins (here moonlighting proteins) it is effective to select a counterpart of the protein group (non-moonlighting proteins) and compare between them as a way of analysing the data. A similar approach has been taken by Frishman et al. in analysing protein-protein interactions by constructing a database called Negatome, which contains protein pairs that are unlikely to physically interact [64] and also in functional analysis of proteins by constructing a database called NoGO, which is a database of proteins that are unlikely to have certain GO terms [65]. Although there is undeniable possibility that some proteins in the non-moonlighting protein dataset may be found to be moonlighting in future, the analysis using the non-moonlighting protein dataset gave conclusion at this point, which can serve as workable hypotheses for future research as scientific works are desired to do. Moreover, we believe that the procedure to select non-moonlighting proteins is quite reasonable: They are proteins which have a sufficient number of GO term annotations but do not have as many functionally distinct terms as known moonlighting proteins.*

*Having written our opinion above in response to the reviewer’s comment, we consider it as an important point. Therefore, we to clarify our standpoint, added following sentences in Discussion: p. 25: “The characteristics of moonlighting proteins were investigated by comparing their features with those of non-moonlighting proteins. In general, finding examples that do not possess a certain property is not straightforward as future research may find that the examples actually do have the property. So are non-moonlighting proteins – there is an undeniable possibility that non-moonlighting proteins used in this study will be found as moonlighting in the future. Nevertheless we believe the current research is valuable and has contributed in progressing our understanding of moonlighting proteins since the non-moonlighting proteins were selected in a reasonable way and also because the differences and similarities of characteristics of moonlighting non-moonlighting proteins were clarified that can serve as hypotheses in the future works. We would also like to point out that similar approaches of selecting negative data sets were taken in analyzing protein-protein interactions (by constructing a non-interacting protein dataset, Negatome [*64*]) and also in analyzing proteins with particular functions (by constructing the NoGo database [*65*]).”*

Indeed, the trends in protein-protein interactions reported in this paper are mostly weak and uninformative.

Authors’ response: *The trends in protein-protein physical interactions of moonlighting proteins are very clear. The number of clusters of interacting proteins for moonlighting proteins is significantly larger than non-moonlighting proteins as the statistical test shows (Supplementary Table S1, Figures. 5B and 5C). Also, Figure 6C shows that interacting proteins of a moonlighting protein are clearly dominated with the moonlighting proteins’ primary function. The trends of co-expressed genes, phylogenetically related proteins, and genetic interactions of moonlighting proteins are weak. However, we think knowing the indifference of moonlighting and non-moonlighting proteins is also useful for understanding moonlighting proteins because this is the first time that moonlighting proteins are analysed in various aspects in comparison with non-moonlighting proteins.*

Attempts to mine the GO database in order to identify new moonlighting proteins are of greater interest but then, again, what is the status of novelty here if this cases can be validated through the published literature?

Authors’ response: *Since this is the first work that proposes a procedure of identifying moonlighting proteins, we needed to show that the proposed procedure (clustering GO terms using the semantic similarity score) can indeed find moonlighting proteins by confirming with the literature. Of course one can read literature of all the genes in an organism to find moonlighting proteins. But the proposed automatic procedure has significantly reduced the amount literature to read. Thus, the proposed computational procedure is an effective filter to identify potential moonlighting proteins. The proposed computational procedure in this work would also trigger development of fully-automated or semi-automated procedure for identifying novel moonlighting proteins that needs none or minimum effort of reading literature in the future.*

*The validation step with published literature also provided insights about situations when proteins selected by the proposed procedure are not moonlighting, i.e. when proteins with distinct GO terms are not actually moonlighting.*

At best, this analysis can help to systematize the data on multiple protein functions. And, the authors do not do a careful job in this systematic survey as one can immediately see from Table 1 that is supposed to present major results of the study. For instance, aspartokinase/homoserine dehydrogenase (for some reason, listed twice in the table) is not a moonlighting protein, it is simply a two-domain bifunctional proteins. There is a difference that the authors seem not to recognize.

Authors’ response: *The two aspartokinase/homoserine dehydrogenase are aspartokinase/homoserine dehydrogenase I and II. To clarify, we added gene IDs, ThrA and MetL. In the revised manuscript, we separated multi-domain multi-functional proteins to a separate table, Table 2 and accordingly, ThrA and MetL are now moved to Table**2**.*

The activity of CysG as a methyltransferase and syroheme synthase are one and the same, this i simply a confusion about terms.

Authors’ response: *The description of this protein’s function in UniProt is “Multifunctional enzyme that catalyzes the SAM-dependent methylations of uroporphyrinogen III at position C-2 and C-7 to form precorrin-2 via precorrin-1. Then it catalyzes the NAD-dependent ring dehydrogenation of precorrin-2 to yield sirohydrochlorin. Finally, it catalyzes the ferrochelation of sirohydrochlorin to yield siroheme.” Based on this UniProt description, we changed functions of CysG to more detailed ones (Function 1: SAM-dependent methylation; Function 2. NAD-dependent ring dehydrogenation; Ferrorochelation).*

Aconitases (aconitate hydratase in the article) seem to present a good example of moonlighting but this is by no account new, the dual role of these proteins had been studied for decades.

Authors’ response: *Aconitase is listed as moonlighting proteins for four organisms in in the MoonProt database (H. Sapiens, M. Tuberculosis, S. Cerevisiae, and B. Taurus) and from three organisms in MultitaskProtDB (H. Sapiens, M. Tuberculosis, S. Cerevisiae) but aconitase of E. coli (AcnA and AcnB) are not included yet. Since ortholog of moonlighting proteins are not necessarily moonlighting, we thought it was worthwhile to list aconitase of E. coli.*

For many proteins, e.g. transporters, involvement in stress response is hardly evidence of moonlighting because they employ their intrinsic activity. One could continue through the entire table as more or less every entry is confusing. One again, bioinformatic study of moonlighting is not at all easy. Unfortunately, I do not think the authors of this paper stand up to the challenge.

Authors’ response: *We agree that levels of experimental evidence of selected moonlighting proteins differ. That is the reason why we have classified the identified potential moonlighting proteins into three categories, I to III (I is the most clear moonlighting proteins, and III is the weakest), according to the level of certainty based on available experimental evidence. The cases pointed out by the reviewer are classified to category III. Thus it is possible that the pointed out cases are found to be non-moonlighting once the molecular mechanism of all function of the proteins are revealed.*

Not for publication but important: the manuscript is sloppy. There are many non-grammatical sentences, typos, quite a few references are incomplete etc.

Quality of written English: Needs some language corrections before being published

Authors’ response: *We made language corrections with help by a native English speaker.*

### Reviewer 2 (Second Round): Dr. Eugine Koonin

The authors have provided reasonable and informative responses to the points made in my review, so i refrain from making further substantive points.

Authors’ response: *Thank you.*

However, I have spotted and corrected several typos in my review, and furthermore, for some reason, all the punctuation except for periods has disappeared from the text of the review. The corrected version is below.

Corrected version of the review:

Khan et al. report an extensive computational analysis of "moonlighting proteins". They correctly note that bioinformatic study of such proteins presents a difficult challenge. Much to my regret, I do not actually believe that the present manuscript meets the challenge to enhance the existing understanding of the moonlighting phenomenon. Moonlighting is not easy to define: it is one of the situations that are rather typical in biology where the "classic" examples are clear and compelling (see crystallins) but few and far between, whereas away from the spotlight matters become fuzzy. I am actually inclined to think that all proteins perform multiple roles in organisms and are at some level moonlighting. The reasons why we think of some but not other proteins as moonlighting have to do mostly with the level of our knowledge and the feasibility of defining discrete functional roles for a given protein. Consequently, I am deeply skeptical about the validity of the control set of "non-moonlighting" proteins and about any comparative analyses that attempt to contrast properties of moonlighting and non-moonlighting proteins.

Indeed, the trends in protein-protein interactions reported in this paper are mostly weak and uninformative.

Attempts to mine the GO database in order to identify new moonlighting proteins are of greater interest but then again, what is the status of novelty here if these cases can be validated through the published literature?

At best this analysis can help to systematize the data on multiple protein functions.And the authors do not do a careful job in this systematic survey as one can immediately see from Table 1 that is supposed to present major results of the study. For instance, aspartokinase/homoserine dehydrogenase (for some reason, listed twice in the table) is not a moonlighting protein, it is simply a two-domain, bifunctional protein. There is a difference that the authors seem not to recognize.

The activity of CysG as a methyltransferase and syroheme synthase are one and the same, this is simply a confusion about terms.

Aconitases (aconitate hydratase in the article) seem to present a good example of moonlighting but this is by no account new as the dual role of these proteins had been studied for decades.

For many proteins e.g. transporters involvement in stress response is hardly evidence of moonlighting because they employ their intrinsic activity.

One could continue through the entire table as more or less every entry is confusing.

Once again, bioinformatic study of moonlighting is not at all easy. Unfortunately, I do not think the authors of this paper stand up to the challenge.

Authors’ response: *As the reviewer pointed out, we found that the commas were dropped somehow when we copied the reviewer’s comments to the manuscript. We put them back to the text of the review. We apologize for the mistake.*

Quality of written English: Acceptable

### Reviewer 3 (First Round): Professor Nick Grishin (University of Texas Southwestern Medical Center and Howard Hughes Medical Institute, Texas, USA)

In this well-executed study, the authors investigate the possibility of a systematic computational approach to find proteins that possess more than one function. Difficulties and advances along their path are discussed. Several general conclusions and nice examples are presented. This work is performed carefully and appears solid. Hopefully, these computational approaches will mature enough to be used by biologists in a quest for new functions of old proteins.

Authors’ response: *Thank you.*

A general, and more philosophical, comment is about the definition of "function" and "moonlighting." The authors already expanded the term to include enzymes that can perform somewhat different reactions. Should "moonlighting" further be expanded to cover multidomain proteins, in which different domains have different functions, or should the term be applied only to a single domain that has several functions? I am asking because if different functions are conveyed by different segments of the polypeptide chain, it might be difficult to distinguish these possibilities without a careful case-by-case study. i.e., disordered segment that carries a different function may be thought of as a separate "domain," and such a protein will not be moonlighting. Basically, if we expand the definition, almost every protein will be moonlighting. If we make the definition more stringent: i.e., the same evolutionary domain with at least two very different functions, maybe moonlighting would be a very rare exception. These comments are not meant as a critique of this excellent study, but just an invitation for thought.

Authors’ response: *Thank you for this very important comment. As the reviewer pointed out, multi-domain multi-function proteins add intriguing complexity in investigating moonlighting proteins. According to the definition of moonlighting proteins proposed by Jeffrey, one of the pioneers of studying moonlighting proteins, proteins with multiple function due to gene fusion are excluded from moonlighting proteins. However, in her opinion multi-domain multi-functional proteins that did not gain multiple domains by gene fusion during evolution (i.e. multi-domain proteins from the beginning) are included in moonlighting proteins (personal communication). Thus, defining moonlighting proteins can be complicated.*

*In this revision, we have simply removed multi-domain proteins from moonlighting proteins as we responded to Reviewer 1’s comment. Multiple-domain proteins are now separately handled in the analyses (figures) and multi-domain domain proteins in E. coli that were originally listed as moonlighting in Table**1**are now moved to a new Table**2**as multi-domain multi-functional proteins.*

## Additional file

Additional file 1: Table S1. P-value from Kolmorov-Smirnov (KS) test for clustering profiles. The table lists the p-values of KS tests performed in the clustering profile analyses.

## Abbreviations

GO: Gene Ontology
PFP: Protein Function Prediction
ESG: Extended Similarity Group
PPI: Protein-protein interaction
TCA: Tri-carboxylic acid
MF: Molecular function
CC: Cellular component
BP: Biological process
KS: Kolmogorov-Smirnov
MP: Moonlighting protein
DLD: Dihydrolipoamide dehydrogenease
FAD: Flavin adenine dicucleotide
NAD: Nicotinamide adenine dinucleotide
VEGF: Vascular endothelial growth factor
SSrel: Relevance semantic similarity score
