# Supplementary material for: Genome-scale identification and characterization of moonlighting proteins
Source: Biol Direct. 2014 Dec 11;9:30. doi: 10.1186/s13062-014-0030-9 (PMC4307903; doi:10.1186/s13062-014-0030-9)
Supplement: Additional file 1: Table S1. — P-value from Kolmorov-Smirnov (KS) test for clustering profiles. The table lists the p-values of KS tests performed in the clustering profile analyses. [file 13062_2014_30_MOESM1_ESM.docx]

# Supplementary Information for

**Genome-Scale Identification and Characterization of Moonlighting Proteins**

Ishita Khan, Yuqian Chen, Tiange Dong, Xioawei Hong, Rikiya Takeuchi, Hirotada Mori, & Daisuke Kihara

Department of Biological Sciences, Department of Computer Science, Purdue University

Contact: [dkihara@purdue.edu](mailto:dkihara@purdue.edu)

**Supplementary Table S1.** P-value from Kolmorov-Smirnov Test for Clustering Profiles

For the clustering profiles shown as figures, the Kolmorov-Smirnov test was performed to examine if the number of clusters formed at specified cutoff is significantly different between a moonlighting protein dataset (MPR1, 2, 3, or the *E. coli* MP set) and *E. coli* non-moonlighting protein set. Refer to corresponding figure captions and text.

| **Dataset** | | **MP sets compared with the *E. coli* non-MP set** | | | |
| --- | --- | --- | --- | --- | --- |
| **Description of data** | **Score Cutoff** | **MPR1** | **MPR2** | **MPR3** | **E. coli MP** |
| Number of BP GO term clusters grouped with *SS^rel^* (Fig. 4A) | 0.1 | **< 0.05** | **< 0.05** | **< 0.05** | **< 0.05** |
|  | 0.5 | **< 0.05** | **< 0.05** | **< 0.05** | **< 0.05** |
|  | 1.0 | **< 0.05** | **< 0.05** | **< 0.05** | **< 0.05** |
| Number of MF GO term clusters grouped with *SS^rel^* (Fig. 4B) | 0.1 | **< 0.05** | **< 0.05** | 0.37 | 0.10 |
|  | 0.5 | 0.07 | 0.12 | 0.10 | 0.25 |
|  | 1.0 | **< 0.05** | **< 0.05** | 0.09 | **< 0.05** |
| Number of clusters of interacting proteins grouped with funsim (Fig. 5B) | 0.2 | 0.61 | 0.14 | 0.60 | 0.16 |
|  | 0.6 | 0.96 | 0.93 | **< 0.05** | **< 0.05** |
|  | 0.8 | **< 0.05** | **< 0.05** | **< 0.05** | **< 0.05** |
| Number of clusters of interacting proteins grouped with BP-funsim (Fig. 5C) | 0.2 | 0.42 | 0.33 | 0.16 | **< 0.05** |
|  | 0.6 | 0.89 | 0.69 | **< 0.05** | **< 0.05** |
|  | 0.8 | 0.08 | 0.19 | **< 0.05** | **< 0.05** |
| Number of clusters of coexpressed proteins grouped with funsim (Fig. 7B) | 0.2 | - | - | - | 0.83 |
|  | 0.6 | - | - | - | 0.75 |
|  | 0.8 | - | - | - | 0.38 |
| Number of clusters of coexpressed proteins grouped with BP-funsim (Fig. 7C) | 0.2 | - | - | - | 0.82 |
|  | 0.6 | - | - | - | 0.35 |
|  | 0.8 | - | - | - | 0.17 |
| Number of clusters of phylogenetically related proteins grouped with funsim (Fig. 8B) | 0.2 | 0.07 | 0.59 | 0.26 | 0.27 |
|  | 0.6 | 0.16 | 0.08 | 0.23 | 0.30 |
|  | 0.8 | 0.15 | 0.45 | **< 0.05** | 0.08 |
| Number of clusters of phylogenetically related proteins grouped with BP-funsim (Fig. 8C) | 0.2 | 0.07 | 0.70 | 0.47 | 0.65 |
|  | 0.6 | 0.15 | 0.08 | 0.17 | 0.36 |
|  | 0.8 | 0.11 | **< 0.05** | **< 0.05** | 0.29 |

1. Moonlighting proteins in the MPR1, 2, 3 sets are from various different organisms. Since gene expression data of all the organisms were not available, we omit the MPR1-3 sets from this analysis.
